# Tanshinone IIA delays liver aging by modulating oxidative stress

**DOI:** 10.3389/fphar.2024.1434024

**Published:** 2024-10-02

**Authors:** Qi Liu, Xu Li, Yi Luo

**Affiliations:** ^1^ Department of Cardiology, Sir Run Run Shaw Hospital, School of Medicine, Zhejiang University, Hangzhou, China; ^2^ School of Basic Medicine, Zhejiang University, Hangzhou, China; ^3^ Research Center for Life and Health Sciences, Binjiang Research Institute, Zhejiang University, Hangzhou, China

**Keywords:** Tanshinone IIA, liver, aging, ESRRG, CYP2E1

## Abstract

Organ-specific aging is increasingly recognized for its research significance, with liver aging demonstrating particular relevance due to its central role in metabolism. We have pioneered the discovery that the expression of ESRRG in the liver positively correlates with age and have established its association with clinical characteristics, including hepatic edema. Our findings link liver aging to a shift in oxidative stress states, where ESRRG, a crucial nuclear receptor responsive to oxidative stress, may be modulated by various small molecules. Through virtual screening of a natural medicinal molecule database followed by further validation, we confirmed that the natural compound Tanshinone IIA mitigates oxidative stress-induced damage in the liver via the ESRRG/Cyp2e1 pathway, thus decelerating liver aging. Importantly, our study also explores the dynamic impact of Tanshinone IIA on ESRRG conformation, providing a profound understanding of its molecular interactions with ESRRG and laying a foundation for the rational design of small molecules based on natural compounds.

## 1 Introduction

The aging liver presents a complex interplay of biochemical processes and regulatory mechanisms that significantly impact overall metabolic health and longevity (Schmucker, 1998; Pinto et al., 2020; Azman et al., 2021; Wang et al., 2024; Amin et al., 2020; Allaire and Gilgenkrantz, 2020; Gieseler et al., 2023). As an essential organ, the liver’s ability to maintain homeostasis diminishes with age, characterized by a decline in regenerative capacity and an increase in oxidative stress (Azman et al., 2021; Bellanti and Vendemiale, 2021; Yang et al., 2019; Cheng et al., 2017; Sastre et al., 2003). This phenomenon necessitates a deeper exploration into the specific factors that contribute to hepatic senescence.

One of the pivotal elements in this context is the modulation of oxidative stress, a critical factor accelerating liver aging (Bellanti and Vendemiale, 2021; Sastre et al., 2003; Cogger et al., 2004; Sanchez-Roman and Barja, 2013). The oxidative stress state in the liver changes dynamically with age, influencing cellular integrity and function. ESRRG (Estrogen-Related Receptor Gamma) emerges as a key player in this scenario (Jung et al., 2024; Vernier et al., 2020; Fan et al., 2018; Billon et al., 2023; Alaynick et al., 2007; Rangwala et al., 2010). Known as an orphan nuclear receptor, ESRRG binds to estrogen-related response elements (ERRE) to promote the transcription of downstream genes involved in the oxidative stress response, thus playing a crucial role in sensing and adjusting to oxidative stress levels (Han et al., 2016).

The exploration of natural small molecules with antioxidant properties presents a promising avenue for modulating ESRRG activity and, consequently, mitigating oxidative stress-induced hepatic aging (Zheng et al., 2023; Badirujjaman et al., 2023; Giner et al., 2022; Charlton et al., 2023; Ribaudo and Gianoncelli, 2023; Malik et al., 2021). Although numerous natural compounds are known for their antioxidative capabilities, the specific molecular targets and mechanisms through which they act remain largely unexplored. This gap in knowledge underscores the necessity of identifying small molecules that can specifically interact with and modulate ESRRG as a means of delaying or reversing liver aging.

## 2 Materials and methods

### 2.1 Animal study

Male C57BL/6N mice, sourced from SLAC (Shanghai, China), were utilized to examine the age-dependent effects of Tanshinone IIA (TAS) on health biomarkers. The study incorporated two age groups: young mice (2 months old) and aged mice (18 months old), with each group consisting of 12 mice. These mice were randomly assigned to two dietary groups: one receiving standard chow and the other receiving chow supplemented with TAS.

The TAS-enriched diet was prepared by integrating 7 g of Tanshinone IIA (Sigma-Aldrich, St. Louis, MO, United States) into 1 kg of standard rodent chow to ensure a homogeneous distribution of the compound throughout the feed. The control group received an equivalent diet without TAS. Mice were fed their respective diets *ad libitum* for 6 weeks under controlled environmental conditions, which included a 12-h light/dark cycle, with water freely available at all times.

Body weight was monitored weekly, and general health was assessed throughout the study. At the end of the feeding period, mice were euthanized under deep anesthesia using isoflurane, and tissues were collected for subsequent biochemical analyses.

### 2.2 Cell culture and treatment

AML12 cells, obtained from ATCC, were cultured in DMEM/F-12 medium enriched with 40 ng/mL dexamethasone and 1% insulin-transferrin-selenium-pyruvate (Gibco, Grand Island, NY, United States), and maintained at 37°C in a 5% CO₂ atmosphere. To establish an *in vitro* model for oxidative stress, the cells were treated with incremental concentrations of hydrogen peroxide (H₂O₂; 0.1 mM, 0.2 mM, 0.3 mM, 0.4 mM, 0.5 mM; Sigma-Aldrich, St. Louis, MO, United States) for varying durations (2 h, 6 h, 12 h, 24 h). Cell viability was quantitatively assessed using the Cell Counting Kit-8 (CCK-8; Dojindo Molecular Technologies, Rockville, MD, United States), and reactive oxygen species (ROS) generation was measured by dihydroethidium (DHE) staining.

Following this initial modeling, cells were also exposed to different concentrations of Tanshinone IIA (TAS; 2 μM, 5 μM, 10 μM, 20 μM, 50 μM; A10031,Yuanye, Shanghai,China) for 24 h to determine the optimal concentration for enhancing cell survival and mitigating ROS production. Based on these results, cells were pre-treated with 10 µM TAS or an equivalent volume of DMSO as a control for 24 h prior to exposure to 0.2 mM H₂O₂ for 4 h, confirming that this regimen maintains cell viability while inducing ROS. This method establishes a controlled, reproducible system to study the antioxidative effects of TAS in response to oxidative stress in liver cells.

### 2.3 Evaluation of TAS-induced antioxidative effects in AML12 hepatocyte oxidative stress model

AML12 hepatocytes were propagated and subsequently exposed to Tanshinone IIA (TAS) according to an established *in vitro* protocol aimed at examining the compound’s influence on reactive oxygen species (ROS) production following hydrogen peroxide (H₂O₂) challenge. Following the treatment with TAS, cells were incubated with the fluorescent probe dihydroethidium (DHE; S0063, Beyotime, Shanghai, China) at a concentration of 10 µM for 30 min at 37°C in an environment shielded from light. DHE is known for its sensitivity to ROS, reacting to form a red-fluorescent product, which facilitates the detection and quantification of oxidative activity within the cells.

The fluorescence emanating from the reacted DHE was assessed using a Leica Dmi3000 fluorescence microscope. Subsequently, images captured were analyzed quantitatively with ImageJ software (National Institutes of Health, Bethesda, MD, United States). The intensity of the DHE fluorescence, serving as an indirect measure of ROS levels, was quantified to evaluate the antioxidative properties of TAS on hepatocytes subjected to oxidative stress.

### 2.4 β-galactosidase staining for assessing cellular senescence in AML12 cells

AML12 cells were cultured under standard conditions and subsequently treated with Tanshinone IIA (TAS) to explore its effects on hydrogen peroxide (H₂O₂)-induced senescence, following a well-established *in vitro* protocol. Post-treatment, the cells were fixed using 4% formaldehyde for 15 min at room temperature. To assess senescence-associated β-galactosidase activity, cells were stained with a solution containing 1 mg/mL 5-bromo-4-chloro-3-indolyl β-D-galactoside (C0602, Beyotime, Shanghai, China), 5 mM potassium ferrocyanide, and 5 mM potassium ferricyanide, at pH 6.0. The staining procedure was carried out at 37°C for 16 h, allowing detailed visualization of senescence markers.

For imaging, the cells were examined under a bright-field microscope, which enabled the capture of detailed cellular morphology. Quantification of β-galactosidase-positive cells was conducted using ImageJ software (National Institutes of Health, Bethesda, MD, United States). This analysis provided a percentage of senescent cells, which helped determine the protective or inducing effects of TAS on cellular senescence.

### 2.5 Measurement of mitochondrial membrane potential in AML12 cells using TMRE staining

AML12 cells were cultured under controlled conditions and treated with Tanshinone IIA (TAS) following an established *in vitro* protocol to investigate its effect on mitochondrial membrane potential. After exposure to TAS, cells were incubated with 200 nM tetramethylrhodamine ethyl ester (TMRE; C2001S, Beyotime, Shanghai, China) for 30 min at 37°C in the dark. TMRE is a cell-permeant dye that accumulates in active mitochondria and is used to assess mitochondrial depolarization, a key indicator of mitochondrial dysfunction.

The level of mitochondrial depolarization was quantified by measuring the fluorescence intensity using a fluorescence microscope. This quantification enabled the assessment of mitochondrial membrane potential changes that are reflective of mitochondrial health or dysfunction.

Data analysis was conducted using ImageJ software (National Institutes of Health, Bethesda, MD, United States). The analysis included quantifying the fluorescence intensity from multiple independent experiments to statistically evaluate the protective effects of TAS against hydrogen peroxide-induced mitochondrial damage. This approach ensures a rigorous quantitative assessment of mitochondrial integrity in response to treatment.

### 2.6 Immunohistochemical analysis of γ-H2AX in liver tissue and immunofluorescence analysis of ESRRG in AML12 cells

AML12 cells were cultured and treated with Tanshinone IIA (TAS) as per the established *in vitro* protocol. After TAS exposure, cells were fixed with 4% paraformaldehyde, permeabilized with 0.1% Triton X-100, and blocked using 5% bovine serum albumin. Cells were incubated overnight at 4°C with a primary antibody against Estrogen Related Receptor Gamma (ESRRG; 1:200 dilution, 14017-1-AP, Proteintech, Wuhan, China). This was followed by a 1-h incubation at room temperature in the dark with an Alexa Fluor 488 conjugated secondary antibody (1:500 dilution, srb2GCL488-1, Proteintech, Wuhan, China). Nuclei were stained with 4′,6-diamidino-2-phenylindole (DAPI; Sigma-Aldrich, St. Louis, MO, United States). Imaging was performed using fluorescence microscopy, and ImageJ software (National Institutes of Health, Bethesda, MD, United States) was used for quantitative analysis.

For the *in vivo* experiments, young (2-month-old) and aged (18-month-old) mice were fed a diet supplemented with Tanshinone IIA (TAS), consistent with previous dietary protocols used in our *in vivo* model. Liver tissues were collected post-treatment, fixed in 10% buffered formalin, and embedded in paraffin. Sections (5 µm) were prepared, deparaffinized, rehydrated, and subjected to antigen retrieval using sodium citrate buffer (pH 6.0). Blocking was performed with 10% normal goat serum, followed by overnight incubation at 4°C with an anti-γ-H2AX antibody (1:200; GB111841-100, Servicebio, Wuhan, China). After incubation with a biotinylated secondary antibody (1:500 dilution; RGAR011, Proteintech, Wuhan, China) for 1 h at room temperature, sections were treated with streptavidin-HRP (Horseradish Peroxidase) and developed with DAB (3,3′-diaminobenzidine) substrate (Sigma-Aldrich, St. Louis, MO, United States). Counterstaining was performed with hematoxylin, and the slides were mounted for microscopic examination. The images were analyzed using ImageJ software for quantification of γ-H2AX positive cells.

### 2.7 qPCR analysis of oxidative stress gene expression in ESRRG-overexpressed AML12 cells

To investigate the transcriptional alterations of oxidative stress-related genes in AML12 cells overexpressing mouse Estrogen Related Receptor Gamma (ESRRG) after exposure to Tanshinone IIA (TAS), quantitative polymerase chain reaction (qPCR) was utilized. AML12 cells were cultured in DMEM/F-12 medium supplemented with 40 ng/mL dexamethasone and 1% insulin-transferrin-selenium-pyruvate under the same conditions as other cell types. Cells underwent transient transfection with a plasmid encoding mouse ESRRG (Baimaike, Beijing, China) using Lipofectamine 3,000 (Thermo Fisher Scientific, Waltham, MA, United States), following the manufacturer’s protocol.

Forty-eight hours post-transfection, total RNA was extracted using the RNeasy Mini Kit (Qiagen, Hilden, Germany). RNA quality and quantity were determined using a NanoDrop spectrophotometer (Thermo Fisher Scientific, Waltham, MA, United States). From 1 µg of total RNA, complementary DNA (cDNA) was synthesized utilizing the High-Capacity cDNA Reverse Transcription Kit (Applied Biosystems, Foster City, CA, United States).

qPCR was conducted on a QuantStudio 5 Real-Time PCR System (Applied Biosystems, Foster City, CA, United States) with SYBR Green PCR Master Mix (Applied Biosystems, Foster City, CA, United States). The thermal cycling conditions included an initial denaturation at 95°C for 10 min, followed by 40 cycles of 95°C for 15 s, and 60°C for 1 min. Primers specific for Cyp1a1, Cyp1a2, Cyp1b1, Cyp2e1, Hif1a, Nox1, Nox4, Sod2, Cat, and Ahr are listed in Supplementary Table 1. Relative gene expression levels were calculated using the 2^-ΔΔCT method, with β-actin as the internal control.

Data were analyzed using the statistical software integrated with the QuantStudio 5 system, and results were expressed as fold changes in gene expression relative to control cells transfected with an empty vector.

### 2.8 Luciferase reporter assay for assessing ESRRG regulation of ERREs in AML12 cells

To assess the regulatory impact of overexpressed Estrogen Related Receptor Gamma (ESRRG) on the activity of wild-type, deletion, and mutant Estrogen-Related Receptor Elements (ERREs) upstream of the mouse Cyp2e1 gene, a series of luciferase reporter assays were conducted. Reporter constructs encompassing the wild-type ERRE sequence, a deletion of this sequence, or mutations within the sequence were engineered and cloned into the pGL3 vector system (Promega, Madison, WI, United States) by Baimaike Biotechnology Co., Ltd.

AML12 cells (mouse hepatocyte cell line, ATCC) were cultured in DMEM/F-12 medium supplemented with 40 ng/mL dexamethasone and 1% insulin-transferrin-selenium-pyruvate under the same conditions as other cell types. The transfection regimen in 24-well plates included co-transfection of 500 ng of the respective luciferase reporter construct with 500 ng of a plasmid encoding mouse ESRRG or an equivalent amount of empty vector to ensure uniform DNA levels across all experimental groups. The transfections were executed using Lipofectamine 3,000 (Thermo Fisher Scientific, Waltham, MA, United States), adhering to the manufacturer’s protocols.

For transfection efficiency normalization, each well was also co-transfected with 20 ng of a Nano-Glo luciferase vector (Promega, Madison, WI, United States) serving as an internal control. Forty-8 h post-transfection, luciferase activities were quantified utilizing the Dual-Luciferase Reporter Assay System (Promega, Madison, WI, United States). Firefly luciferase measurements were normalized against Nano luciferase outputs to evaluate the effects of ESRRG overexpression on the transcriptional activity driven by the different ERRE constructs under the influence of Tanshinone IIA (TAS).

### 2.9 Measurement of liver function biomarkers in mice treated with tanshinone IIA

To evaluate the effects of Tanshinone IIA (TAS) on liver function indicators across different age groups of mice, measurements were taken for albumin (ALB), total protein (TP), alanine aminotransferase (ALT), aspartate aminotransferase (AST), and alkaline phosphatase (ALP). Blood samples were collected from the orbital sinus under isoflurane anesthesia 24 h following the final administration of TAS. Serum was obtained by centrifugation at 3,000 rpm for 10 min and analyzed using a fully automated clinical chemistry analyzer (VetScan VS2, Abaxis, Union City, CA, United States), which was specifically calibrated for mouse serum. Reagents and control samples for ALB, TP, ALT, AST, and ALP were sourced from Abaxis (Union City, CA, United States).

All experimental procedures adhered to ethical guidelines for animal care and were approved by the Institutional Animal Care and Use Committee. This method allows for the reliable quantification of hepatic biomarkers, providing insights into the hepatoprotective or hepatotoxic potential of Tanshinone IIA in mice, thereby reflecting its impact on liver health across various life stages.

### 2.10 Flow cytometric analysis of ROS production in AML12 cells treated with Tanshinone IIA

To evaluate the effect of Tanshinone IIA (TAS) on reactive oxygen species (ROS) production in AML12 cells, we followed a previously established *in vitro* experimental protocol. The cells were treated with TAS and subsequently incubated with 10 µM 2′,7′-dichlorodihydrofluorescein diacetate (S0033M, Beyotime, Shanghai, China) for 30 min at 37°C in a dark environment to assess ROS levels. The DCFH-DA probe is metabolized into the fluorescent compound 2′,7′-dichlorofluorescein (DCF) upon oxidation by ROS, which serves as an indicator of cellular oxidative stress.

Following the incubation period, the cells were washed and resuspended in phosphate-buffered saline (PBS), then immediately analyzed by flow cytometry (BD LSRFortessa, BD Biosciences, San Jose, CA, United States). Fluorescence was recorded at an excitation wavelength of 485 nm and an emission wavelength of 528 nm. Data analysis was conducted using FlowJo software (BD Biosciences, San Jose, CA, United States), with a focus on the mean fluorescence intensity (MFI) of DCF, reflecting the concentration of intracellular ROS.

### 2.11 Molecular dynamics simulations of ESRRG-Tanshinone IIA interactions

Molecular dynamics (MD) simulations of the Estrogen Related Receptor Gamma (ESRRG) in both null and Tanshinone IIA-bound (TAS) forms were conducted to explore structural dynamics and interaction energies. The simulations were performed using the GROMACS simulation package, version 2020.4 (www.gromacs.org), employing the AMBER14sb force field.

Initial structures of ESRRG-Apo and ESRRG-TAS complexes were modeled based on their respective crystal structures. Missing residues were added, and hydrogen atoms were assigned according to the AMBER14sb force field specifications. Each system was solvated in an explicit TIP3P water box with a minimum padding of 10 Å from any edge of the protein to the box boundary. Sodium and chloride ions were added to achieve electroneutrality and simulate physiological ionic strength.

Energy minimization was performed using the steepest descent method to eliminate any steric clashes or inappropriate geometries. This was followed by an 80 ns MD simulation at a constant temperature (300 K) and pressure (1 atm), using a V-rescale thermostat and a Berendsen barostat. The Particle Mesh Ewald (PME) method was utilized for long-range electrostatic interactions, and all bonds involving hydrogen atoms were constrained using the LINCS algorithm.

Trajectory analyses, including root mean square deviation (RMSD), root mean square fluctuation (RMSF), and hydrogen bond analysis, were conducted post-simulation to evaluate the stability and conformational changes of the protein complexes. These results offer insights into the molecular mechanisms of ESRRG interaction with TAS, enhancing our understanding of its role in signaling pathways.

### 2.12 Virtual screening of TCMSP compounds targeting ESRRG using molecular docking

To identify potential inhibitors of Estrogen Related Receptor Gamma (ESRRG) from the Traditional Chinese Medicine Systems Pharmacology Database (TCMSP), we employed an integrated computational approach. The active binding pockets on ESRRG were initially identified using DogSiteScorer from the ProteinPlus web server (ProteinPlus, Berlin, Germany).

Compounds from TCMSP were prepared for docking simulations using Open Babel (Open Babel Software Foundation) to optimize the structures and convert them into the appropriate docking formats. These compounds, including Tanshinone IIA (TAS), were then docked into the predicted active sites of ESRRG using AutoDock Vina, enhanced with GPU acceleration to improve the speed and efficiency of the docking process (Ding et al., 2023).

Post-docking, the binding affinities of the compounds were analyzed using RDKit (RDKit: Open-source cheminformatics). Additionally, ADMETlab 3.0 (ADMETlab, Zhejiang University, China) was utilized to predict the absorption, distribution, metabolism, excretion, and toxicity (ADMET) properties of the top-ranking ligands. This comprehensive analysis assessed each compound’s drug-likeness and potential as a therapeutic agent.

Visualization of the docking poses and interaction patterns was conducted using PyMOL (Schrödinger, LLC, New York, NY, United States) for molecular graphics and Matplotlib (Matplotlib Development Team) for the quantitative display of the results. This visualization provided a detailed examination of the interactions between ESRRG and potential inhibitors, including TAS, offering insights into the binding mechanisms.

### 2.13 Western blot analysis of ESRRG and CYP2E1 in TAS-treated AML12 cells

Following treatment with Tanshinone IIA (TAS) in established *in vitro* models, AML12 cells were lysed using RIPA buffer (Thermo Fisher Scientific, Waltham, MA, United States) supplemented with protease and phosphatase inhibitors (Sigma-Aldrich, St. Louis, MO, United States). The lysates were centrifuged at 14,000 × g for 15 min at 4°C, and the protein concentration in the supernatant was determined using a BCA Protein Assay Kit (Pierce Biotechnology, Rockford, IL, United States). Proteins (30 µg per sample) were separated on a 10% SDS-PAGE and transferred to PVDF membranes (Bio-Rad Laboratories, Hercules, CA, United States). The membranes were blocked with 5% non-fat dry milk in TBST for 1 h at room temperature and incubated overnight at 4°C with primary antibodies against ESRRG (1:1,000, 14017-1-AP, Proteintech, Wuhan, China) and CYP2E1 (1:1,000, 19937-1-AP, Proteintech, Wuhan, China). Following primary antibody incubation, membranes were treated with horseradish peroxidase-conjugated secondary antibodies (1:5,000, SA00001-2, Proteintech, Wuhan, China) for 1 h at room temperature. Bands were visualized using enhanced chemiluminescence (ECL, GE Healthcare, Chicago, IL, United States) and quantified with ImageJ software (NIH, Bethesda, MD, United States).

### 2.14 Biochemical analysis of antioxidant enzymes and oxidative stress markers in TAS-treated liver tissues

Liver tissues were finely minced and homogenized in saline to create a 10% w/v solution, followed by centrifugation at 3,000 g for 10 min at 4°C. The supernatant from the tissue homogenate was used to determine the total antioxidant capacity (T-AOC) and the activity levels of key antioxidant enzymes after treatment with Tanshinone IIA (TAS). Catalase (CAT) activity was measured using the ammonium molybdate spectrophotometric method, superoxide dismutase (SOD) activity was assessed via the hydroxylamine method, and glutathione peroxidase (GPx) levels were determined by a colorimetric method. Additionally, the malondialdehyde (MDA) content, an indicator of lipid peroxidation, was evaluated. All assays were performed using commercially available kits provided by Nanjing Jiancheng Bioengineering Institute (Nanjing, China), following the manufacturer’s instructions. Data were normalized and expressed as units per milligram of protein to ensure comparability across samples, with protein concentrations in the supernatants quantified to adjust enzyme activity measurements.

### 2.15 Ethics statement

All experimental procedures adhered to the ethical guidelines for animal care, as stipulated by the Animal Ethics Committee of The Second Affiliated Hospital, School of Medicine, Zhejiang University. The study was conducted following the principles outlined in the Guide for the Care and Use of Laboratory Animals (8th edition, National Research Council). Special attention was given to minimizing animal suffering and distress throughout the study. All animals were housed in a controlled environment with appropriate temperature, humidity, and ventilation, and they were monitored daily for signs of discomfort or illness.

Prior to the initiation of the study, the experimental protocol was reviewed and approved by the Animal Ethics Committee, ensuring that the study design met all ethical requirements for the humane treatment of animals. Mice were euthanized under deep isoflurane anesthesia to ensure a painless and humane endpoint, followed by tissue collection for biochemical analyses. All efforts were made to reduce the number of animals used and to refine the experimental procedures to enhance animal welfare.

### 2.16 Statistical analysis

The R statistical software (v4.3.2) (http://www.r-project.org) was used for statistical calculations. Data were expressed as means ± standard deviation (SD). Multiple comparisons were performed by one-way ANOVA followed by Bonferroni correction. A value of *p* < 0.05 was considered as statistically significant.

## 3 Result

### 3.1 Age-related shifts in liver transcriptome highlight oxidative stress links

**FIGURE 1:**
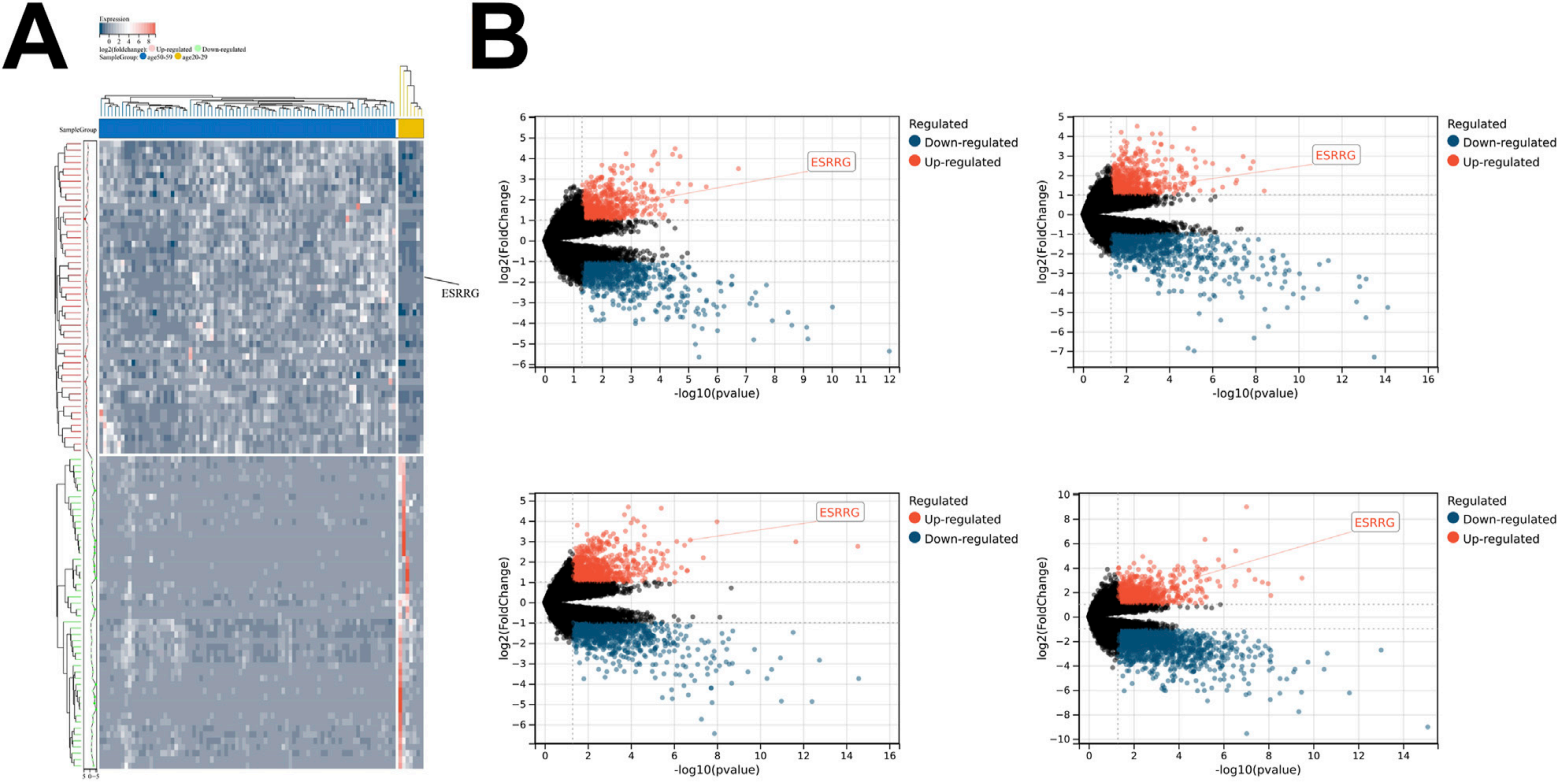
Age-Related Differences in Gene Expression in Liver Transcriptomes **(A)** Heatmap illustrating differentially expressed genes (DEGs) between liver transcriptomes of individuals aged 70–79 and those aged 20–29. Genes are color-coded based on their expression levels, with upregulated genes shown in red and downregulated genes in green. The sample groups are displayed along the top of the heatmap, with the color bar indicating age groups. **(B)** Volcano plots depicting differential gene expression between liver transcriptomes from various age groups compared to those aged 20–29, with upregulated genes in red and downregulated genes in blue. The panels represent the following age groups: 60–69 (top left), 50–59 (top right), 40–49 (bottom left), and 30–39 (bottom right). The *x*-axis represents -log10 (*p*-value), and the *y*-axis represents log2 (fold change). Significant differentially expressed genes, such as ESRRG, are annotated. Statistical significance is indicated by the thresholds in the plots.

**FIGURE 2:**
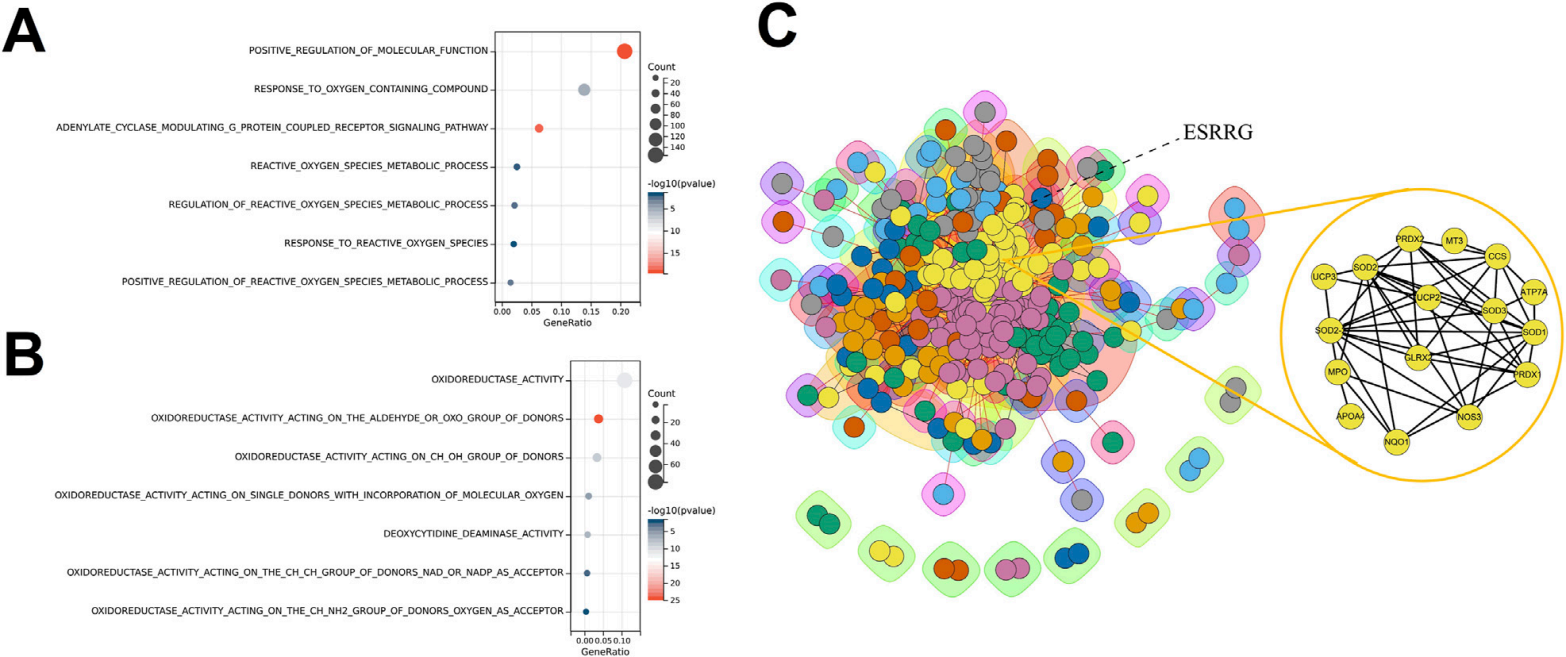
Enrichment Analyses and Protein-Protein Interaction Network for Age-Specific Differential Gene Expression in Liver Transcriptomes **(A)** Biological Process (BP) enrichment analysis for differentially expressed genes (DEGs) between liver transcriptomes of individuals aged 70–79 and those aged 20–29, showing significant enrichment in processes such as positive regulation of molecular function and reactive oxygen species metabolic process. **(B)** Molecular Function (MF) enrichment analysis for DEGs between liver transcriptomes of individuals aged 70–79 and those aged 20–29, highlighting functions related to oxidoreductase activity and deoxyribonucleotide metabolism. **(C)** Protein-Protein Interaction (PPI) network constructed from DEGs between liver transcriptomes of individuals aged 70–79 and those aged 20–29, with clusters of interconnected proteins involved in critical metabolic and regulatory pathways. ESRRG is identified as a key node, indicating its central role in the network, particularly in association with oxidative stress and lipid oxidation processes.

Transcriptomic analysis of liver tissues across different age groups, based on data from the GTEx database, revealed that ESRRG expression was significantly upregulated in the age groups 30–39, 40–49, 50–59, 60–69, and 70–79 years compared to the 20–29 age group (Figures 1A, B). Enrichment analysis of differentially expressed genes (DEGs) between the 70–79 and 20–29 years groups identified significant enrichment in biological processes related to the reactive oxygen species metabolic process, highlighting the role of oxidative stress in aging (Figure 2A). Additionally, molecular function analysis showed significant enrichment in terms associated with oxidoreductase activity, implicating a prominent role in redox reactions (Figure 2B).

The protein-protein interaction (PPI) network constructed from DEGs positioned ESRRG at a central node, with clustering analysis revealing strong associations with oxidative stress and lipid oxidation processes (Figure 2C). Gene set variation analysis (GSVA) comparing the transcriptomes of the 60–79 and 20–39 age groups showed that most identified transcription factors, including the crucial oxidative regulator HIF1a, were predominantly suppressed in the older group (Supplementary Figure 1A). Interestingly, biological processes such as regulation of cell aging and positive regulation of reactive oxygen species metabolic process were significantly enriched, underscoring the link between organ aging and oxidative stress (Supplementary Figure 1B).

### 3.2 WGCNA links ESRRG with age-related liver pathology and oxidative stress indicators

**FIGURE 3:**
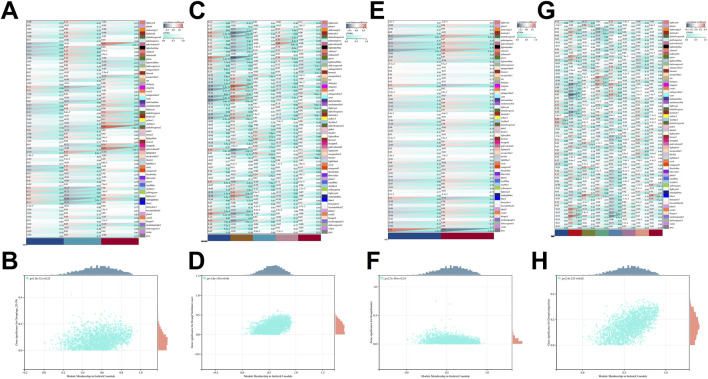
WGCNA-Identified gene module correlations with clinical and pathological characteristics. **(A)** Correlation of WGCNA-identified gene modules with age-related traits. **(B)** Scatter plot showing GS-MM correlation for the firebrick3 module with age characteristics (20–39 years). **(C)** Correlation of WGCNA-identified gene modules with mortality traits. **(D)** Scatter plot showing GS-MM correlation for the firebrick3 module with mortality traits (ventilator case). **(E)** Correlation of WGCNA-identified gene modules with gender characteristics. **(F)** Scatter plot showing GS-MM correlation for the firebrick3 module with gender characteristics (female). **(G)** Correlation of WGCNA-identified gene modules with pathological traits. **(H)** Scatter plot showing GS-MM correlation for the firebrick3 module with pathological characteristics (congestion).

In the analysis of liver tissue transcriptomics, ESRRG was located within the firebrick3 module identified by weighted gene co-expression network analysis (WGCNA) (Supplementary Figure 2). This module’s correlation with clinical features was explored, revealing a marginally significant association with the age group 20–39 years (p-value = 0.06) and a correlation coefficient of 0.13. The firebrick3 module showed no significant correlation with gender. In relation to mortality characteristics, the module displayed significant correlations with intermediate death (coefficient = 0.17), ventilator case (coefficient = 0.33), and natural causes (coefficient = −0.35). Additionally, this module was linked to liver pathology, specifically showing a negative correlation with congestion (coefficient = −0.26) (Figure 3).

**FIGURE 4:**
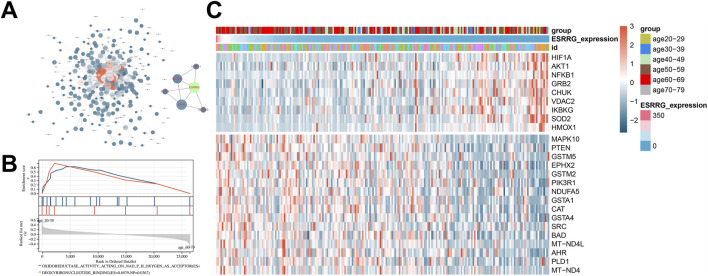
Network analysis and pathway enrichment in liver transcriptomes **(A)** Protein-Protein interaction (PPI) network constructed from genes within the firebrick3 module, emphasizing a subnetwork that includes ESRRG as a central node. **(B)** Gene Set Enrichment Analysis (GSEA) showing enriched molecular function (MF) pathways in liver transcriptomes when comparing age groups 20–39 and 60–79 years. Notable pathways include those related to oxidoreductase activity and deoxyribonucleotide binding. **(C)** Heatmap illustrating the expression of genes associated with the longevity signaling pathway from KEGG, correlated with ESRRG expression levels in liver transcriptomes. The heatmap displays gene expression variations across different age groups, with a focus on how ESRRG abundance influences the expression of these genes.

A protein-protein interaction (PPI) network constructed for genes within the firebrick3 module highlighted direct connections between ESRRG and nodes such as Hif1a, Slc7a1, and Esr2 (Figure 4A). Gene set enrichment analysis (GSEA) comparing transcriptomes from the 60–79 and 20–39 age groups identified pathways related to superoxide production and redox reactions, suggesting the involvement of oxidative stress in liver aging (Figure 4B). Further, correlational analysis between liver transcriptome ESRRG expression levels and longevity-related genes from the KEGG database showed a significant negative correlation between ESRRG expression and the oxidative stress regulator SOD2 (Figure 4C).

### 3.3 Tanshinone IIA exhibits high affinity for ESRRG and potential antioxidative activity

**FIGURE 5:**
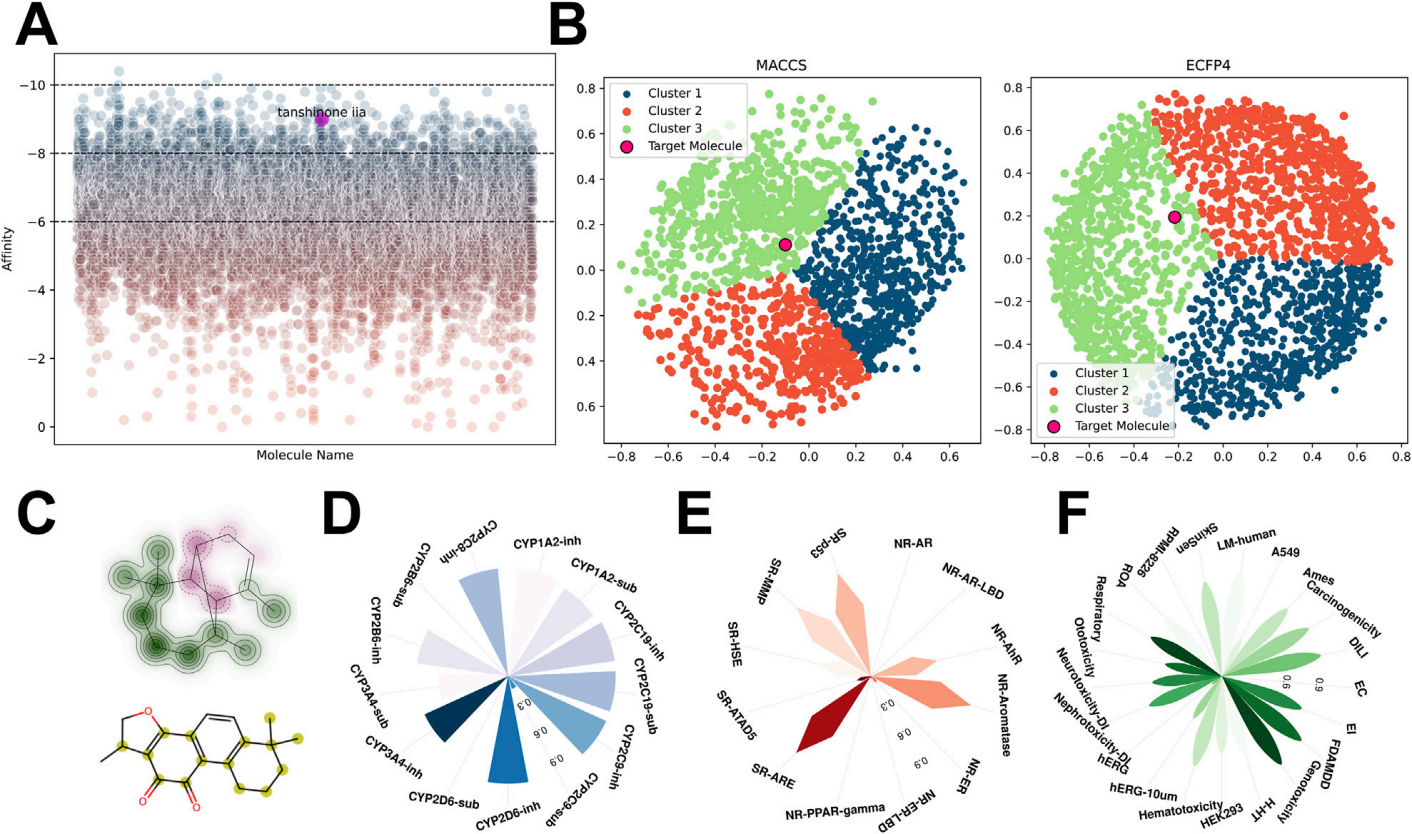
Virtual screening and analysis of compounds targeting ESRRG from the TCMSP database **(A)** Scatter plot depicting the affinity of all compounds for ESRRG identified through virtual screening in the TCMSP database, highlighting tanshinone IIA. **(B)** Clustering of high-affinity small molecules based on MACCS and ECFP4 fingerprints from the TCMSP database virtual screening results, with distinct clusters color-coded. **(C)** Structural similarity of tanshinone IIA (TAS) and its cluster center molecules, with key substructures highlighted. **(D)** Drug metabolism characteristics of tanshinone IIA (TAS) in terms of cytochrome P450 interactions. **(E)** Enrichment analysis of tanshinone IIA (TAS) in the Tox21 database, demonstrating its interaction potential with key nuclear receptors and signaling pathways. **(F)** Toxicity prediction profiles for tanshinone IIA (TAS) across various toxicity endpoints.

Virtual screening of the Traditional Chinese Medicine Systems Pharmacology Database (TCMSP) identified Tanshinone IIA (TAS) as a small molecule with high affinity for the Estrogen Related Receptor Gamma (ESRRG) (Figure 5A). TAS was positioned within the green cluster when clustering analysis was conducted using MACCS and ECFP4 molecular fingerprints (Figure 5B). Structural comparison with cluster center molecules highlights TAS’s substructural similarities, which underpin its classification (Figure 5C).

The planar multi-ring structure of TAS, featuring a five-membered oxygen-containing heterocycle, suggests potential antioxidative properties, given the known activity of oxygenated heterocycles in antioxidative processes. Furthermore, the common pharmacological properties of TAS, including its absorption, distribution, toxicity, metabolism, and enrichment in TOX21 pathways, were thoroughly investigated (Figures 5D–F; Supplementary Figure 3). Notably, TAS’s potential hepatorenal toxicity and genotoxicity were highlighted, along with its activation effects on pathways including SR-ARE, SR-MMP, and SR-p53.

### 3.4 ESRRG binding site characterization and TAS docking dynamics

**FIGURE 6:**
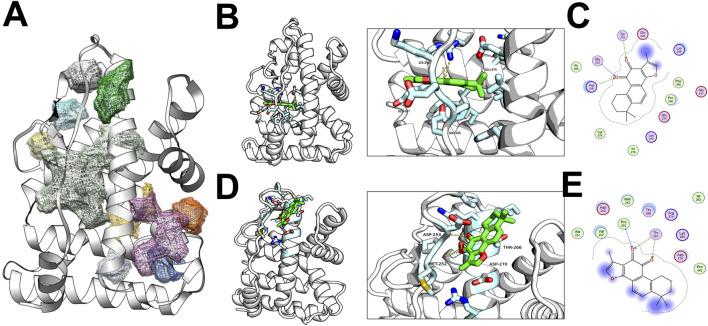
Docking Analysis of TAS with ESRRG ligand binding domain (LBD) **(A)** Distribution of active sites within the ESRRG LBD. **(B)** First class of active site docking between TAS and ESRRG LBD. **(C)** 2D representation of the first class active site docking between TAS and ESRRG LBD. **(D)** Second class of active site docking between TAS and ESRRG LBD. **(E)** 2D representation of the second class active site docking between TAS and ESRRG LBD.

The ligand-binding domain (LBD) of the Estrogen Related Receptor Gamma (ESRRG) was characterized by a conventional anti-parallel α-helical sandwich fold, comprising numerous α-helices (H1-H11) accompanied by a diminutive β-sheet. Within ESRRG, the top ten prospective active sites were identified, with the voluminous light green pocket being localized to the region encircled by H2, H7, and the β-sheet. An additional pair of pockets—one green and the other grey—were situated above the light green pocket, circumscribed by H2 and H5 (Figure 6A).

Docking results revealed Tanshinone IIA (TAS) primarily docked within the light green and green pockets. In the principal docking pose, the small molecule’s active site was partially solvent-exposed, with the occluded aspect forming hydrogen bonds through the oxygen atom on the five-membered ring of TAS with the backbone or side chains of Gly312, Arg316, and Tyr315. Hydrophobic interactions between Ile306, Pro246he, Phe366, Val278, and TAS further stabilized the molecule. The pocket’s contour was also shaped by electrostatic interactions among Glu245, Lys248, Lys370, and Glu275 (Figures 6B, C).

In an alternate major docking pose, the small molecule occupied an active pocket with greater openness. The enclosed side included hydrogen bonds with Pro253, Thr267, and extensive hydrophobic interactions involving Ile262, Met252, Pro445, Val257. The overall pocket configuration was influenced by electrostatic interactions between Asp270, Asp254, Lys263, and Arg274 (Figure 6D, E).

### 3.5 TAS mitigates aging markers in hepatic tissue without impacting liver function

**FIGURE 7:**
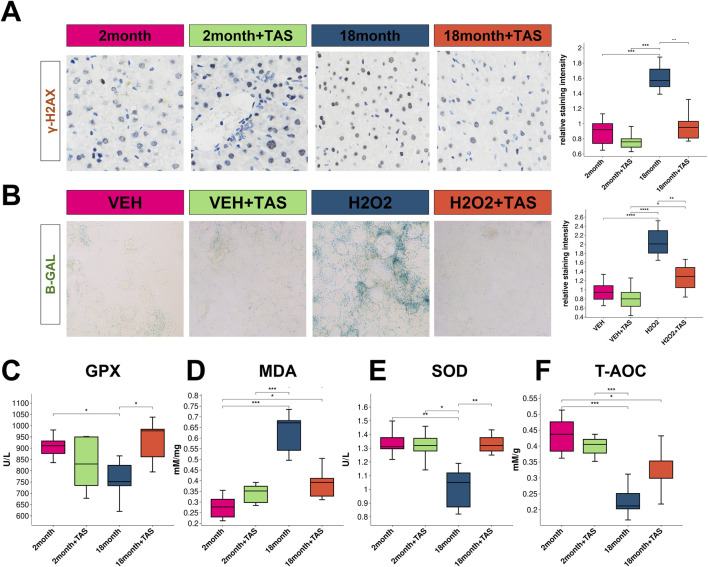
Impact of TAS treatment on aging markers and antioxidant levels in mouse liver **(A)** Immunostaining of the aging marker γ-H2AX in liver sections from different treatment groups: 2-month-old mice, 2-month-old mice treated with TAS, 18-month-old mice, and 18-month-old mice treated with TAS. The staining intensity indicates DNA damage associated with aging, with significant differences observed between groups as shown in the box plot (*p < 0.05, **p < 0.01). **(B)** β-Galactosidase (B-GAL) staining in AML12 liver cells from different treatment groups: VEH (vehicle control), VEH + TAS, H2O2 (hydrogen peroxide treatment), and H2O2 + TAS. The staining highlights cellular senescence, with the relative staining intensity quantified in the box plot (***p < 0.001, **p < 0.01). **(C)** Glutathione Peroxidase (GPX) levels in the liver of mice across different treatment groups: 2-month-old, 2-month-old treated with TAS, 18-month-old, and 18-month-old treated with TAS. The box plot displays significant differences in GPX activity between groups (*p < 0.05). **(D)** Malondialdehyde (MDA) levels, a marker of lipid peroxidation, in the liver of mice from different treatment groups. The box plot shows significant variations across the groups (***p < 0.001, **p < 0.01). **(E)** Superoxide Dismutase (SOD) activity in the liver of mice across different treatment groups, illustrating the changes in oxidative stress defenses (*p < 0.05, **p < 0.01). **(F)** Total Antioxidant Capacity (T-AOC) in the liver of mice from different treatment groups. The box plot indicates significant differences in antioxidant capacity across the treatment groups (***p < 0.001).

Tanshinone IIA (TAS) was observed to mitigate the staining intensity of the aging biomarker γ-H2AX in the liver of aged mice (Figure 7A). Moreover, TAS demonstrated a protective effect *in vitro* against hydrogen peroxide (H₂O₂)-induced hepatocyte aging (Figure 7B). Notably, no significant alterations in liver function were detected between aged and young mice, and TAS exhibited no impact on hepatic function, despite potential hepatotoxicity suggested by prior ADMETlab analyses (Supplementary Figure 4). Significantly, TAS improved markers of glutathione peroxidase (GPX), malondialdehyde (MDA), and superoxide dismutase (SOD) in the livers of aged mice, though it did not affect the total antioxidant capacity (T-AOC) (Figures 7C–F).

### 3.6 TAS modulates oxidative stress response and ESRRG-Cyp2e1 axis in hepatocytes

**FIGURE 8:**
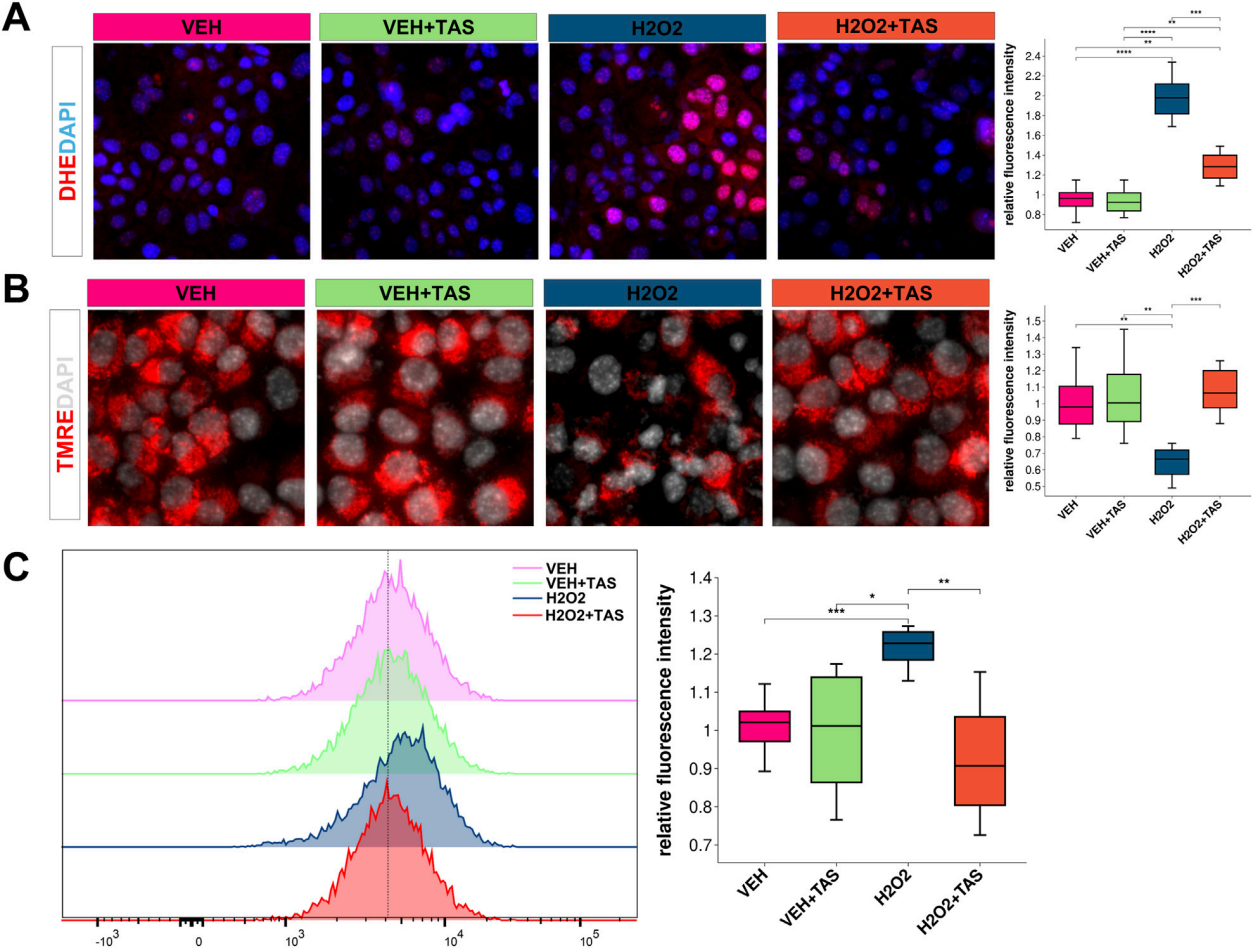
Oxidative Stress and Mitochondrial Membrane Potential in AML12 Cells Under Various Treatments **(A)** DHE staining (red) shows ROS levels in AML12 cells treated with VEH, VEH + TAS, H₂O₂, and H₂O₂ + TAS. Nuclei are stained with DAPI (blue). Quantification of fluorescence intensity per cell is shown (**p < 0.01, ***p < 0.001). **(B)** TMRE staining (red) assesses mitochondrial membrane potential in AML12 cells across the same treatments, with DAPI (gray) for nuclei. Quantified intensity is displayed (**p < 0.01, ***p < 0.001). **(C)** DCFH-DA fluorescence histogram and quantification indicate oxidative stress levels across treatment groups (*p < 0.05, **p < 0.01).

Using dihydroethidium (DHE) staining, we found that Tanshinone IIA (TAS) significantly reduced H₂O₂-induced superoxide production in hepatocytes (Figure 8A). Additionally, TAS preserved mitochondrial membrane potential as demonstrated by TMRE staining (Figure 8B). Flow cytometry with DCFH-DA further confirmed that TAS decreased reactive oxygen species (ROS) generation in these cells (Figure 8C).

**FIGURE 9:**
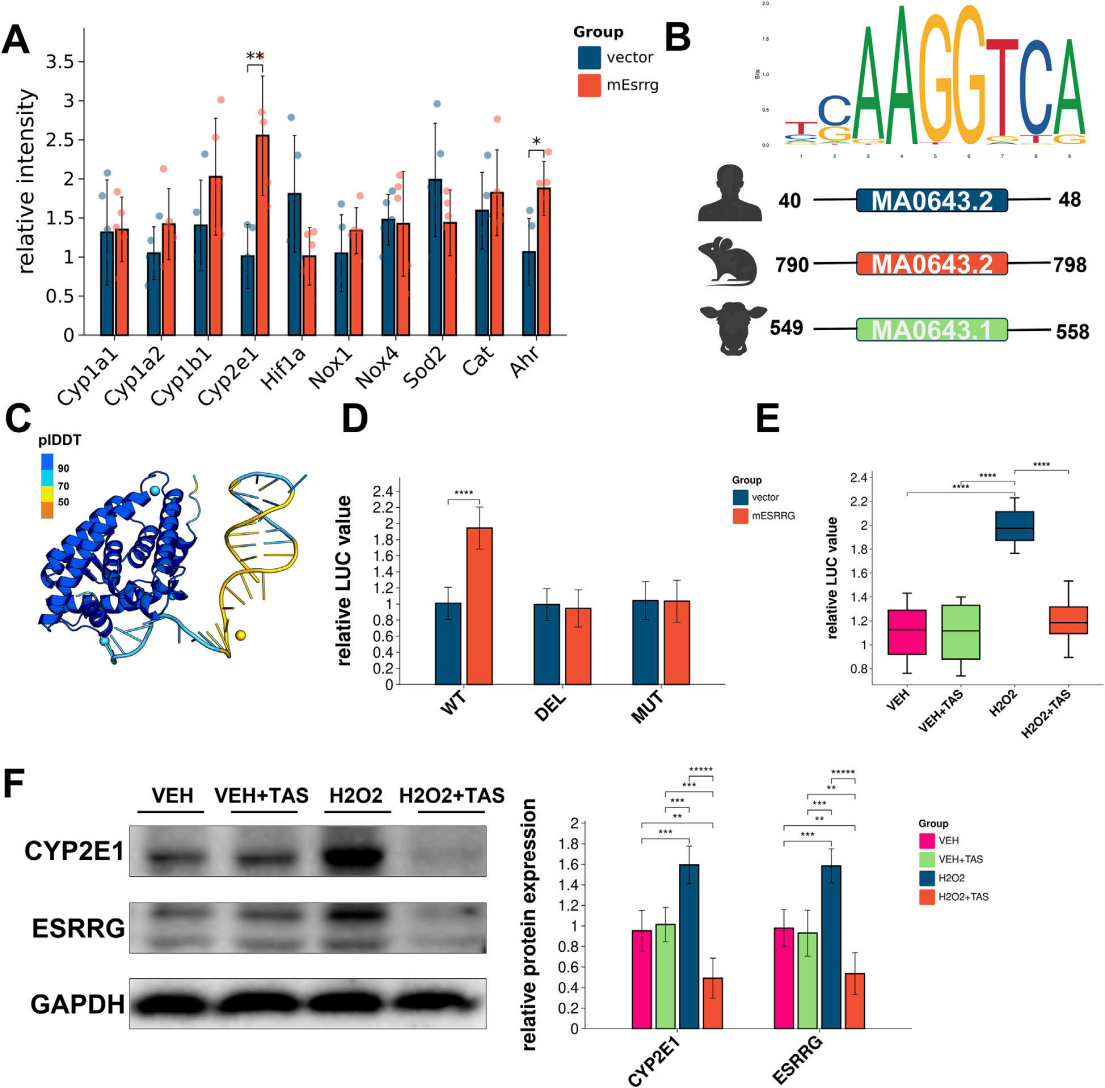
Effects of ESRRG overexpression on oxidative stress response and gene regulation in AML12 Cells **(A)** Transcriptional changes in Ahr and Cyp2e1 genes in AML12 cells following ESRRG overexpression. Significant differences are observed between vector control and mESRRG overexpression, particularly for Ahr and Cyp2e1 (**p < 0.01, *p < 0.05). **(B)** Sequence logo of the ERRE (Estrogen-Related Receptor Response Element) upstream of Cyp2e1 in mouse, human, and cattle, showing the conserved ESRRG binding site. **(C)** Structural model of AF3 depicting the predicted binding of ESRRG to the ERRE motif on the Cyp2e1 promoter. **(D)** Luciferase reporter assay of wild type (WT), deletion (DEL), and mutant (MUT) Cyp2e1 reporters in mice. Significant increases in luciferase activity are seen with mESRRG overexpression in WT (**p < 0.01, ****p < 0.0001). **(E)** Quantification of ESRRG protein levels and localization in AML12 cells under various treatments, showing upregulation and nuclear translocation upon H₂O₂ and H₂O₂+TAS treatment (****p < 0.0001). **(F)** Western blot analysis of Cyp2e1 and ESRRG in AML12 cells under different treatments, normalized to GAPDH. The right panel quantifies relative protein levels (**p < 0.01, ***p < 0.001, ****p < 0.0001).

Gene expression analysis of the reactive oxygen species generation pathway using the KEGG database revealed upregulation of Cyp2e1 and Ahr in hepatocytes following overexpression of mouse ESRRG (Figure 9A). Sequence analysis identified Estrogen-Related Receptor Elements (ERREs) in the promoter regions upstream of Cyp2e1 in humans, mice, and cattle, indicating ESRRG’s potential transcriptional regulation of Cyp2e1 (Figure 9B).

Structural modeling of AF3 predicted interactions between ESRRG and the ERRE motif in the Cyp2e1 promoter, supporting a direct regulatory role (Figure 9C). Luciferase reporter assays in AML12 cells confirmed that ESRRG significantly activated transcription through the wild-type ERRE of Cyp2e1 but failed to do so with deleted or mutated ERREs (Figure 9D). Notably, TAS pretreatment did not suppress Cyp2e1 transcription under basal conditions but did reduce its transcription under oxidative stress induced by H₂O₂ (Figure 9E). Immunofluorescence staining showed that TAS did not affect the nuclear translocation of ESRRG after H₂O₂ treatment (Supplementary Figure 5). Western blotting revealed that TAS exerted a negative regulatory effect on both ESRRG and Cyp2e1 expression, particularly under oxidative stress conditions (Figure 9F).

### 3.7 Impact of TAS binding on ESRRG dynamics

**FIGURE 10:**
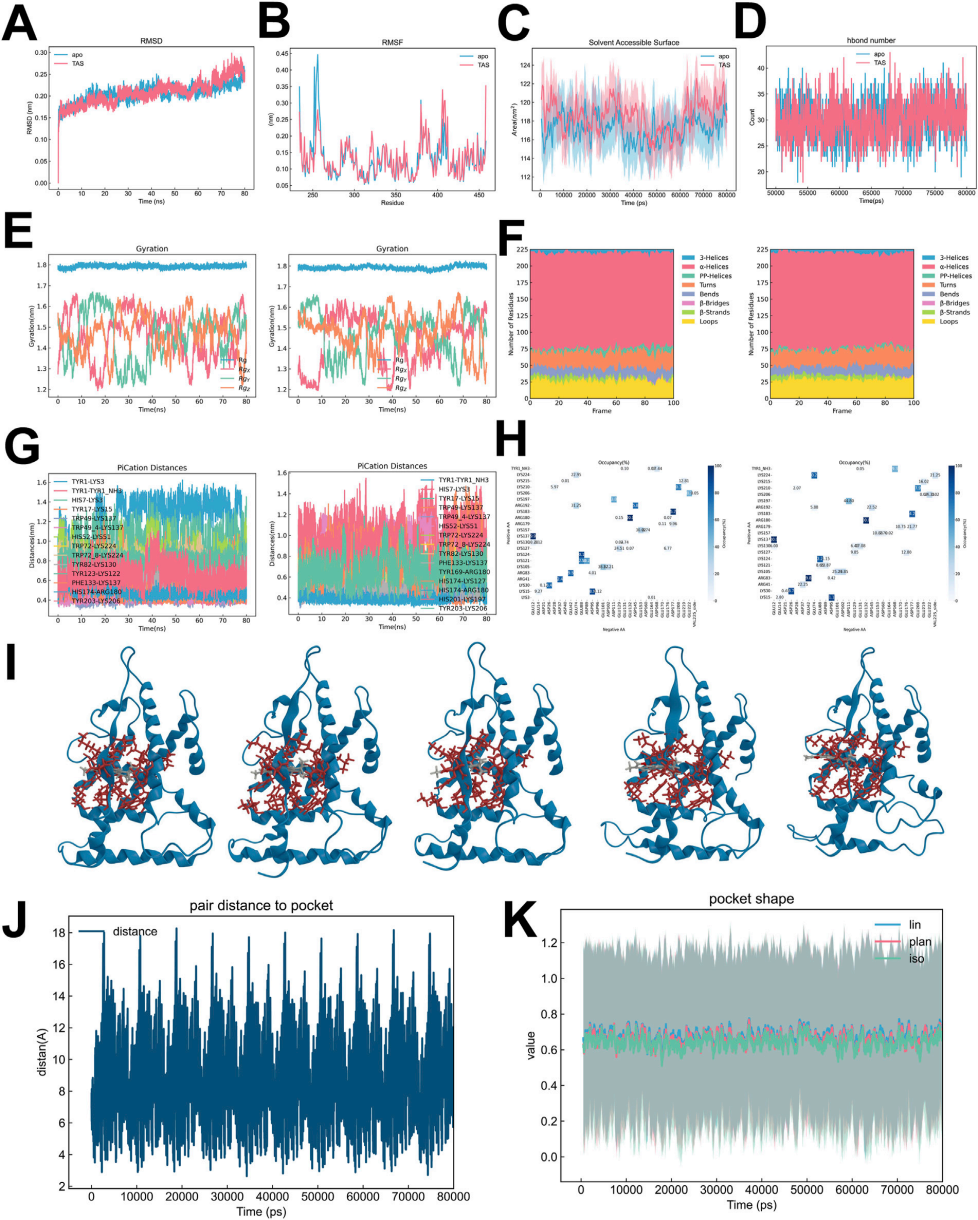
Comparative analysis of ESRRG isoforms in Apo and TAS-bound States **(A)** Root Mean Square Deviation (RMSD) analysis comparing structural deviations over time between ESRRG in the Apo state and when bound to TAS. **(B)** Root Mean Square Fluctuation (RMSF) comparison highlighting the flexibility of specific residues in ESRRG between its Apo state and TAS-bound state. **(C)** Solvent Accessible Surface Area (SASA) analysis showing differences in solvent exposure between ESRRG-Apo and ESRRG-TAS. **(D)** Analysis of the number of hydrogen bonds formed in ESRRG-Apo versus ESRRG-TAS over the simulation time. **(E)** Radius of gyration plots demonstrating the compactness of the ESRRG protein in both Apo and TAS-bound states across multiple replicates. **(F)** Secondary structure distribution analysis using the Dictionary of Secondary Structure of Proteins (DSSP), comparing the structural elements of ESRRG in Apo and TAS-bound states. **(G)** Pi-cation interaction distances measured over time for key residues in ESRRG-Apo and ESRRG-TAS, with specific attention to the interactions critical for TAS binding. **(H)** Occupancy of salt bridges in ESRRG across the simulation frames, comparing the differences between Apo and TAS-bound states. **(I)** Structural snapshots showing the binding pocket of ESRRG at time points 0 ns, 20 ns, 40 ns, 60 ns, and 80 ns during the simulation for both Apo and TAS-bound states. **(J)** Time evolution of pair distances between specific residues and the binding pocket, tracking changes induced by TAS binding in ESRRG. **(K)** Analysis of pocket shape dynamics in ESRRG, comparing the linear, planar, and isotropic values over time between Apo and TAS-bound states.

Molecular dynamics simulations were performed to explore the conformational and functional effects of ESRRG in both ligand-absent (ESRRG-Apo) and Tanshinone IIA-bound (ESRRG-TAS) states over time. Both variants quickly stabilized in terms of root mean square deviation (RMSD), with ESRRG-Apo showing a minor increase towards the end of the simulation (Figure 10A). Detailed analysis revealed similar overall residue fluctuation patterns between ESRRG-Apo and ESRRG-TAS, with localized reductions in fluctuations noted for ESRRG-TAS, particularly in the N-terminal region where the Apo state exhibited higher residue flexibility (Figure 10B). The Solvent Accessible Surface Area (SASA) (Figure 10C) and hydrogen bond count (Figure 10D) were consistent throughout the simulation, indicating that the protein’s exposure to the solvent and the overall hydrogen bonding network remained stable.The radius of gyration analysis (Figure 10E) showed that the overall compactness of ESRRG did not significantly differ between the apo and TAS-bound states. Secondary structure analysis using DSSP (Figure 10F) revealed that the protein maintained its secondary structural elements, with minor fluctuations in helices and bends observed around residue 170 during the 60–80 ns interval. The Pi-cation interaction distances (Figure 10G) were monitored, showing consistent interactions within the active site, which supports the stable binding of TAS. The salt bridge occupancy analysis (Figure 10H) indicated stable ionic interactions, further corroborating the structural integrity of ESRRG throughout the simulation.

We showed the positions of 0ns, 20ns, 40ns, 60ns, and 80ns TAS in the pocket to illustrate their regular movement within the pocket (Figure 10I). The binding pocket analysis indicated that while the pair distance to the pocket boundary showed periodic movements, the overall pocket shape remained stable, as indicated by consistent linear, planar, and isotropic values over time (Figures 10J, K). This suggests that TAS binding does not induce major conformational changes in the binding pocket, preserving its structural integrity.

**FIGURE 11:**
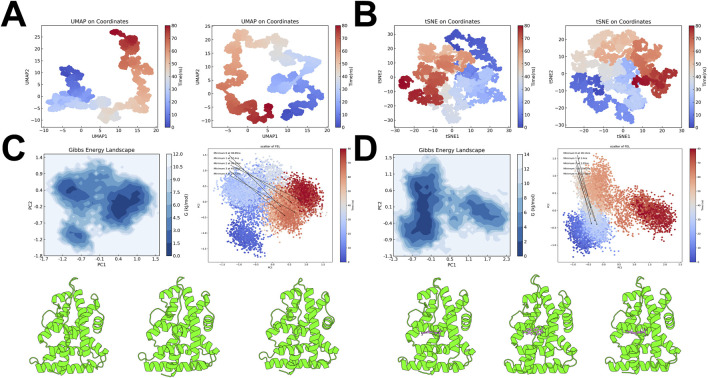
Free Energy Landscape and Key Conformational States of ESRRG Isoforms **(A)** UMAP projections based on atomic coordinates from molecular dynamics simulations, comparing ESRRG in the Apo state (left) and TAS-bound state (right). The color gradient indicates the simulation time, transitioning from blue (early) to red (late). **(B)** t-SNE projections based on atomic coordinates from molecular dynamics simulations, with ESRRG in the Apo state (left) and TAS-bound state (right). The time progression is color-coded from blue to red, reflecting the evolution of conformational states. **(C)** Gibbs Free Energy Landscape (FEL) for ESRRG-Apo, mapped using principal component analysis (PCA). The contour plot highlights the energy minima, with the scatter plot indicating the distribution of conformations over the simulation time. Below the panel, the three lowest energy minima conformations are displayed from left to right. **(D)** Gibbs Free Energy Landscape (FEL) for ESRRG-TAS, similarly mapped using PCA. The contour plot identifies the energy minima, and the scatter plot shows the progression of conformations over time. Below the panel, the three lowest energy minima conformations are displayed from left to right.

UMAP and t-SNE projections based on atomic coordinates from the simulations illustrated the conformational transitions over time. For both ESRRG-Apo and ESRRG-TAS, these projections highlighted the temporal evolution of conformational states, with clear distinctions between early and late simulation stages (Figures 11A, B). The color gradient, transitioning from blue to red, reflects these changes, further supporting the dynamic nature of ESRRG’s response to TAS binding.

To further elucidate the conformational changes induced by TAS binding, the Free Energy Landscape (FEL) was constructed using principal component analysis (PCA). The FEL for ESRRG-Apo showed multiple energy minima, indicating the presence of several stable conformational states, with three low-energy minima identified as the most stable conformations (Figure 11C). In contrast, the FEL for ESRRG-TAS also revealed multiple minima, but with slightly deeper wells and energy minima appearing in the early and mid-simulation stages, suggesting that TAS binding stabilizes distinct conformations earlier in the simulation (Figure 11D).

The analysis based on Residue Distance Correlation Matrices (RDCM) further corroborates these findings. The average distance matrix between residues revealed that the spatial proximity between residue pairs remains relatively consistent across both Apo and TAS-bound states, with some variations in the dynamic interactions (Supplementary Figure 6A). The time occupancy of the contact matrix indicated that residue pairs maintain contact for similar durations in both states, though TAS binding increases contact persistence in certain regions (Supplementary Figure 6B). Interestingly, the DCCM of RDCM did not show significant changes with TAS binding (Supplementary Figure 6C); however, the local correlation patterns between residue pair distances and time were altered (Supplementary Figure 6D). These changes in correlation were accompanied by a shuffling in the hierarchical clustering of residues based on their distance metrics, reflecting a reorganization in residue interactions (Supplementary Figure 6E). The PCA of RDCM demonstrated that by the late stages of the simulation, both Apo and TAS-bound states formed relatively independent clusters, highlighting distinct dynamic behaviors as the simulation progressed (Supplementary Figure 6F).

**FIGURE 12:**
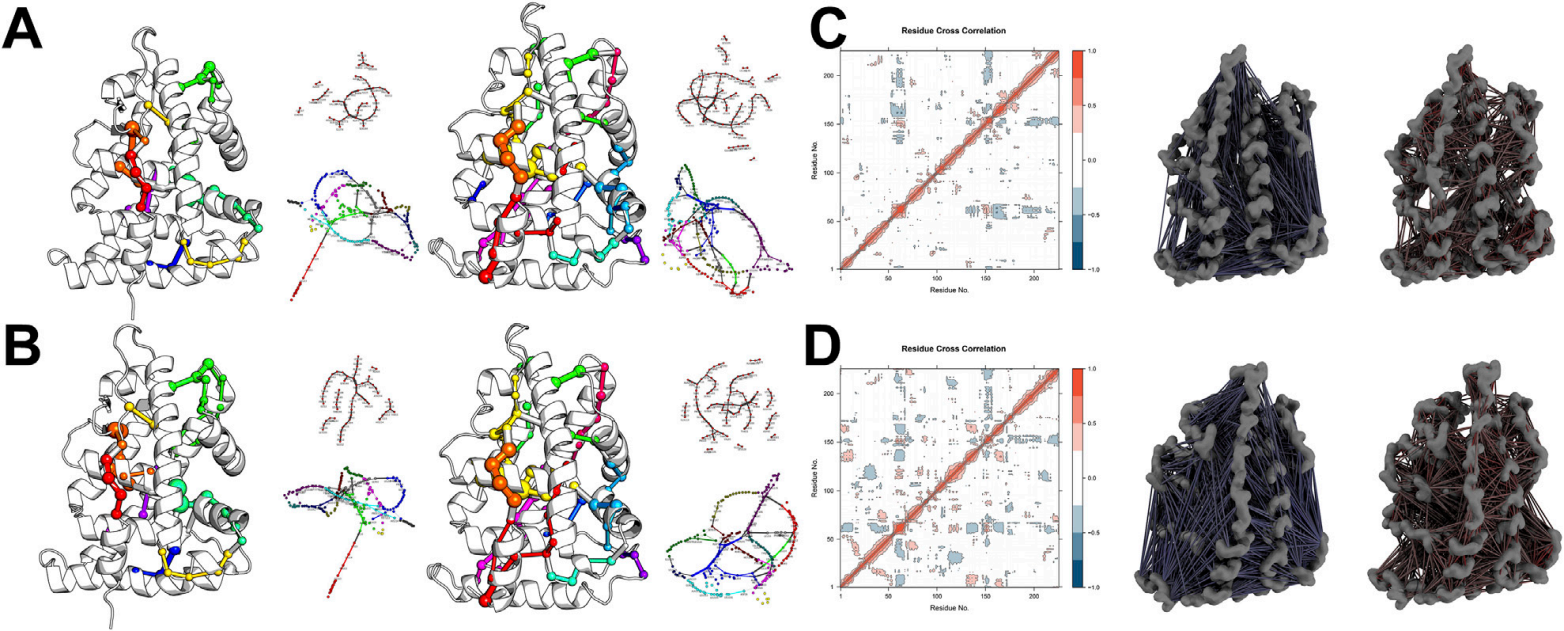
Network pathway analyses and community partitioning in ESRRG structures **(A)** Shortest path network analysis within ESRRG, comparing Apo (left) and TAS-bound (right) states. Nodes are colored to denote different community memberships, while edge thickness represents the weight between nodes calculated based on the dynamic cross-correlation matrix (DCCM). **(B)** Sub-shortest path network analysis within ESRRG, again comparing the Apo (left) and TAS-bound (right) states. Community memberships are indicated by node color, and edge thickness represents the interaction strength between nodes based on DCCM. **(C)** Dynamic Cross-Correlation Matrix (DCCM) and 3D visualization of residue cross-correlations for ESRRG-Apo, indicating antagonistic (blue) and synergistic (red) interactions. **(D)** Dynamic Cross-Correlation Matrix (DCCM) and 3D visualization of residue cross-correlations for ESRRG-TAS, with antagonistic (blue) and synergistic (red) interactions highlighted.

Further network pathway analysis revealed that both the TAS-bound and Apo states of ESRRG display a radial divergence pattern in their residue networks. However, the binding of TAS enhances the structural topology of the two-dimensional residue network, manifested through the extension and establishment of new paths within spatially adjacent secondary structures, particularly helices (Figures 12A, B). While the Dynamic Cross-Correlation Matrix (DCCM) analysis shows that TAS binding does not significantly impact the synergistic movements between adjacent residues along the principal axis, it does increase both the synergistic and antagonistic interactions between residues from different structural elements (Figures 12C, D). This effect is especially pronounced in the N-terminal region, where the Apo state exhibits minimal long-distance residue interactions, a deficiency that is substantially rectified upon TAS binding.

The MM-PBSA calculations for the ESRRG-TAS system resulted in a binding energy value of −63.1038 ± 17.5931 kJ/mol, indicating a stable interaction between TAS and ESRRG. Van der Waals interactions were identified as the primary contributors, with Coulombic and solvation energies also playing significant roles (Supplementary Figure 7A). The energy components (MM, SA, PB) remained stable over time, particularly during the early simulation period from 5 ns to 20 ns (Supplementary Figures 7B–D). Residue-level analysis highlighted that specific residues, particularly around the 40, 80 and 120–140 segments, contributed significantly to the binding energy (Supplementary Figures 7E–G).

## 4 Discussion

The process of aging presents varied rates of decline across different organs, with the liver, as a central organ in metabolic regulation, exhibiting a unique pattern of aging that is crucially influenced by oxidative stress (Tian et al., 2023; Campisi et al., 2019; Rossiello et al., 2022; Coban et al., 2014). Recent research, including our own, suggests that changes in oxidative stress play a significant role in the onset and progression of hepatic aging (Bellanti and Vendemiale, 2021; Coban et al., 2014; Castro et al., 2012). The liver’s pivotal role in detoxifying metabolic byproducts makes its susceptibility to oxidative damage particularly impactful on overall health and longevity (Billon et al., 2023). Our findings build on prior studies that have highlighted the liver’s vulnerability to oxidative stress but extend this understanding by identifying ESRRG as a novel age-related biomarker within the liver, thus providing fresh insights into the molecular dynamics of aging.

We have pioneered the identification of ESRRG’s correlation with aging in the liver, establishing a link between its expression and pathological changes, including congestion. This connection has not been extensively reported in previous studies, marking a significant advancement in understanding the molecular underpinnings of liver aging. In comparison, earlier research primarily focused on more generalized biomarkers without establishing direct correlations with specific age-related changes within the liver (Azman et al., 2021; Cogger et al., 2004; Coban et al., 2014; Castro et al., 2012; Tanikawa and Torimura, 2006; Zhou et al., 2017).

Moreover, the antioxidative properties of various plant-derived small molecules have been well-documented; however, our approach involved a targeted high-throughput screening specifically focusing on ESRRG (Biasutto et al., 2011; Subramanya et al., 2018; Yu et al., 2023). This led to the discovery that Tanshinone IIA, a widely used small molecule, can mitigate liver aging by reducing reactive oxygen species production via the ESRRG/cyp2e1 pathway. This finding not only underscores the potential of Tanshinone IIA in therapeutic applications against liver aging but also highlights a novel interaction that had not been previously explored, distinguishing our results from existing literature.

Furthermore, our in-depth analysis of the binding site and potential conformational changes in ESRRG upon interaction with Tanshinone IIA provides critical insights. By elucidating the molecular interactions between Tanshinone IIA and ESRRG, we have laid the groundwork for drug redesign using natural small molecules as templates. This aspect of our study extends beyond typical pharmacological research by linking biochemical pathways directly to structural pharmacology, a comparative rarity in existing studies, which often do not bridge these two areas as explicitly.

Despite these advancements, there remain several unresolved issues within our study and the field at large. These include the full spectrum of molecular pathways influenced by ESRRG in the context of aging, the long-term effects of Tanshinone IIA on liver health, and the potential for adverse effects when using plant-derived molecules in therapeutic settings (Kim et al., 2016; Yang et al., 2023; Wang et al., 2011).

In conclusion, while our study advances the understanding of liver aging and offers novel therapeutic avenues, it also highlights critical gaps and limitations that must be addressed in future research. These findings not only contribute to the scientific discourse but also pave the way for more effective interventions in the management of liver aging.

## Data Availability

The original contributions presented in the study are included in the article/Supplementary Material, further inquiries can be directed to the corresponding author.

## Supplementary material

SUPPLEMENTARY FIGURE 1 MM-PBSA Energy Calculations for ESRRG-TAS System **(A)** Average MM-PBSA energy components, showing contributions from van der Waals (vdW), Coulombic (cou), molecular mechanics (MM), polar solvation (dPB), and non-polar solvation (dSA) energies. **(B)** Molecular mechanics (MM) energy components (vdW, cou, MM) over time during the simulation. **(C)** Solvent accessible surface area (SA) energy components for the complex, ligand, and protein over time. **(D)** Polar solvation (PB) energy components for the complex, ligand, and protein over time. **(E)** Residue-wise average molecular mechanics energy (MM = cou + vdW) contributions. **(F)** Residue-wise average polar solvation energy (PB) contributions. **(G)** Residue-wise average solvent accessible surface area (SA) energy contributions.

SUPPLEMENTARY FIGURE 2 GSVA Analysis of Transcription Factors and Biological Processes in Liver Transcriptomes **(A)** Gene Set Variation Analysis (GSVA) of transcription factors in liver transcriptomes comparing individuals aged 70–79 to those aged 20–29. **(B)** GSVA of biological processes in liver transcriptomes comparing individuals aged 70–79 to those aged 20–29.

SUPPLEMENTARY FIGURE 3 Comprehensive WGCNA Analyses of Gene and eigengenes Clusters. **(A)** Dendrogram of gene clustering within WGCNA, illustrating hierarchical relationships based on expression patterns. **(B)** Heatmap depicting the clustering of module eigengenes, highlighting the interrelationships among WGCNA modules.

SUPPLEMENTARY FIGURE 4
**(A)** Scatter plot illustrating the absorption properties of TAS across various models. The y-axis lists different in vitro assays, including MDCK (Madin-Darby Canine Kidney cells), caco2 (human colorectal adenocarcinoma cells), PAMPA (Parallel Artificial Membrane Permeability Assay), pgp_sub (P-glycoprotein substrate), pgp_inh (P-glycoprotein inhibitor), hia (human intestinal absorption), and different levels of fraction unbound in plasma (f20, f30, f50). The x-axis represents the log-transformed permeability or interaction values. Larger pink circles indicate stronger absorption or interaction, with green lines representing the range of measurements. **(B)** Distribution profile of TAS across various pharmacokinetic barriers and transporters. The y-axis indicates the predicted values, ranging from 0 to 1, for each factor. The x-axis lists factors including Fu (fraction unbound), PPB (plasma protein binding), BBB (blood-brain barrier penetration), BCRP (Breast Cancer Resistance Protein), MRP1 (Multidrug Resistance-associated Protein 1), BSEP (Bile Salt Export Pump), OATP1B1, OATP1B3 (Organic Anion Transporting Polypeptides), and logVDss (volume of distribution at steady state). Red circles denote significant distribution or interaction, with vertical blue lines representing the range of predictions.

SUPPLEMENTARY FIGURE 5 Liver Function Biomarkers in Mice Exposed to TAS This figure presents the measured levels of liver function indicators: albumin (ALB), alkaline phosphatase (ALP), alanine aminotransferase (ALT), aspartate aminotransferase (AST), and total protein (TP). Each panel corresponds to one of the biomarkers.

SUPPLEMENTARY FIGURE 6 ESRRG Localization in AML12 Cells under Different Treatments Immunofluorescence images of ESRRG (green) and DAPI (blue) in AML12 cells treated with VEH, VEH+TAS, H_2_O_2_, and H_2_O_2_+TAS. The ESRRG signal remains consistently localized to the nucleus across all treatment groups, with no significant changes in localization observed. The right panel quantifies the relative fluorescence intensity per cell across the different treatment groups.

SUPPLEMENTARY FIGURE 7 Analysis of Residue Distances in a Dynamic Matrix Format **(A)** Average distance matrix between residues, showing the mean distances (in nanometers) across the simulation time for each residue pair in the protein. Left: Apo state; Right: TAS-bound state. **(B)** Time occupancy of the contact matrix, indicating the proportion of time during the simulation that each residue pair remains in contact. Left: Apo state; Right: TAS-bound state. **(C)** Dynamic Cross-Correlation Matrix (DCCM) of residue distance correlation matrices (RDCM), displaying the correlation of residue movements over time. Left: Apo state; Right: TAS-bound state. **(D)** Pearson correlation matrix over time, illustrating the time-dependent correlation coefficients between residue pairs. The matrix reveals both positive and negative correlations throughout the simulation. Left: Apo state; Right: TAS-bound state. **(E)** Hierarchical clustering of residues based on distance metrics, revealing groupings of residues that move together during the simulation. Left: Apo state; Right: TAS-bound state. **(F)** Principal Component Analysis (PCA) of the residue distance correlation matrix (RDCM), highlighting the principal components that capture the major modes of movement during the simulation. The scatter plots color-code the time progression from blue (early) to red (late) states. Left: Apo state; Right: TAS-bound state.
