# Supplementary figures and images for: Tanshinone IIA delays liver aging by modulating oxidative stress
Source: Front Pharmacol. 2024 Oct 2;15:1434024. doi: 10.3389/fphar.2024.1434024 (PMC11480062; doi:10.3389/fphar.2024.1434024)

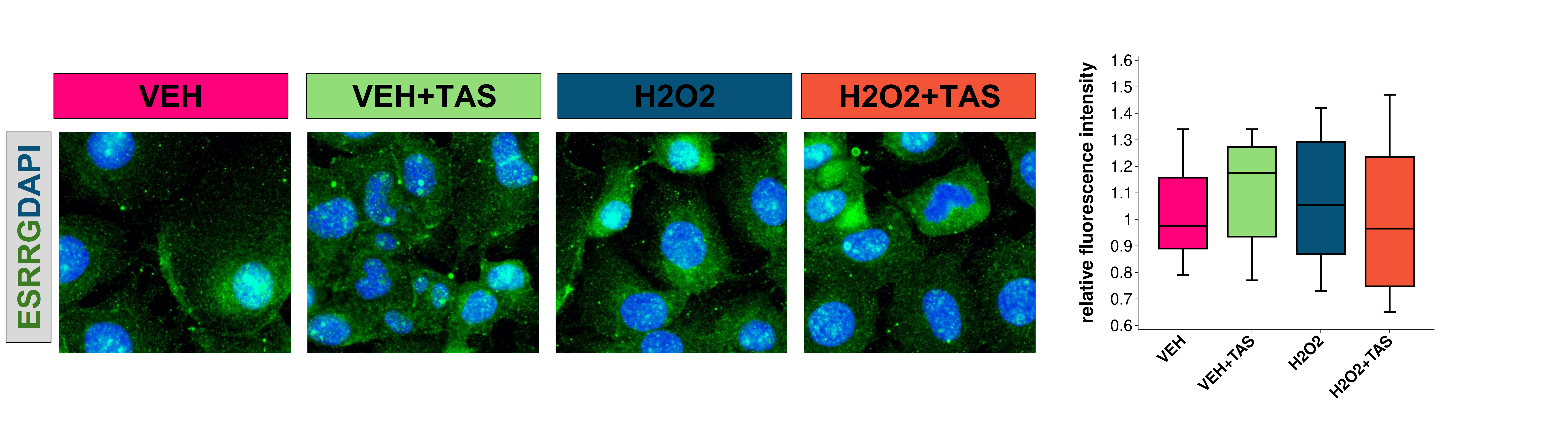

Supplement: Supplementary file 1 [file Image6.TIF]

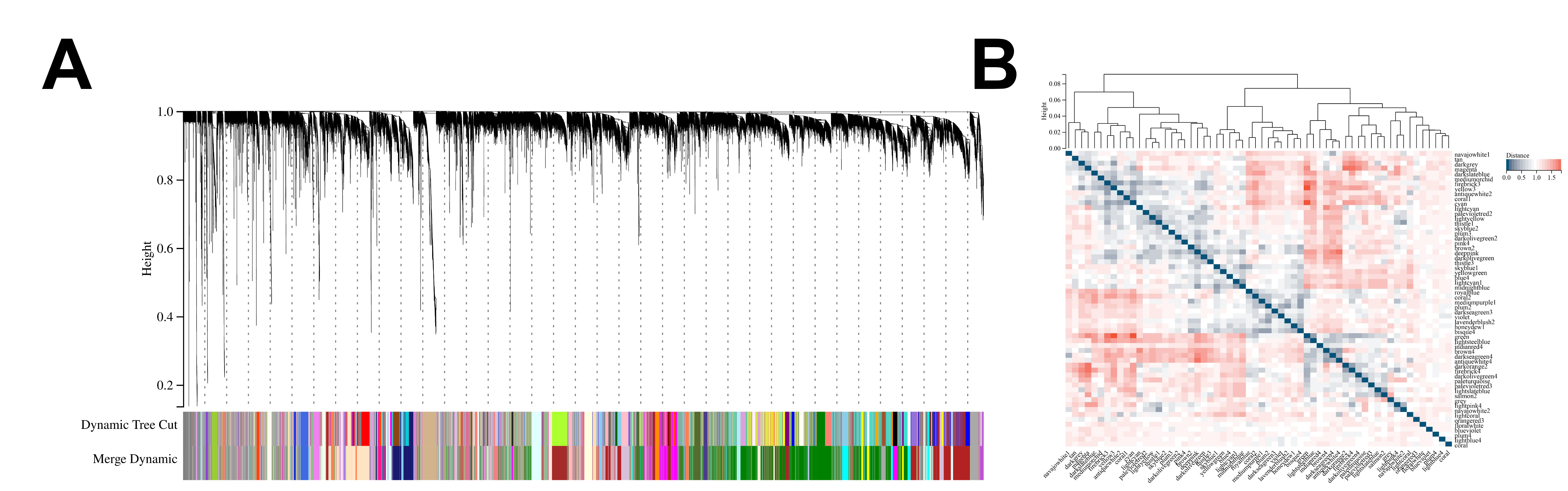

Supplement: Supplementary file 2 [file Image3.TIF]

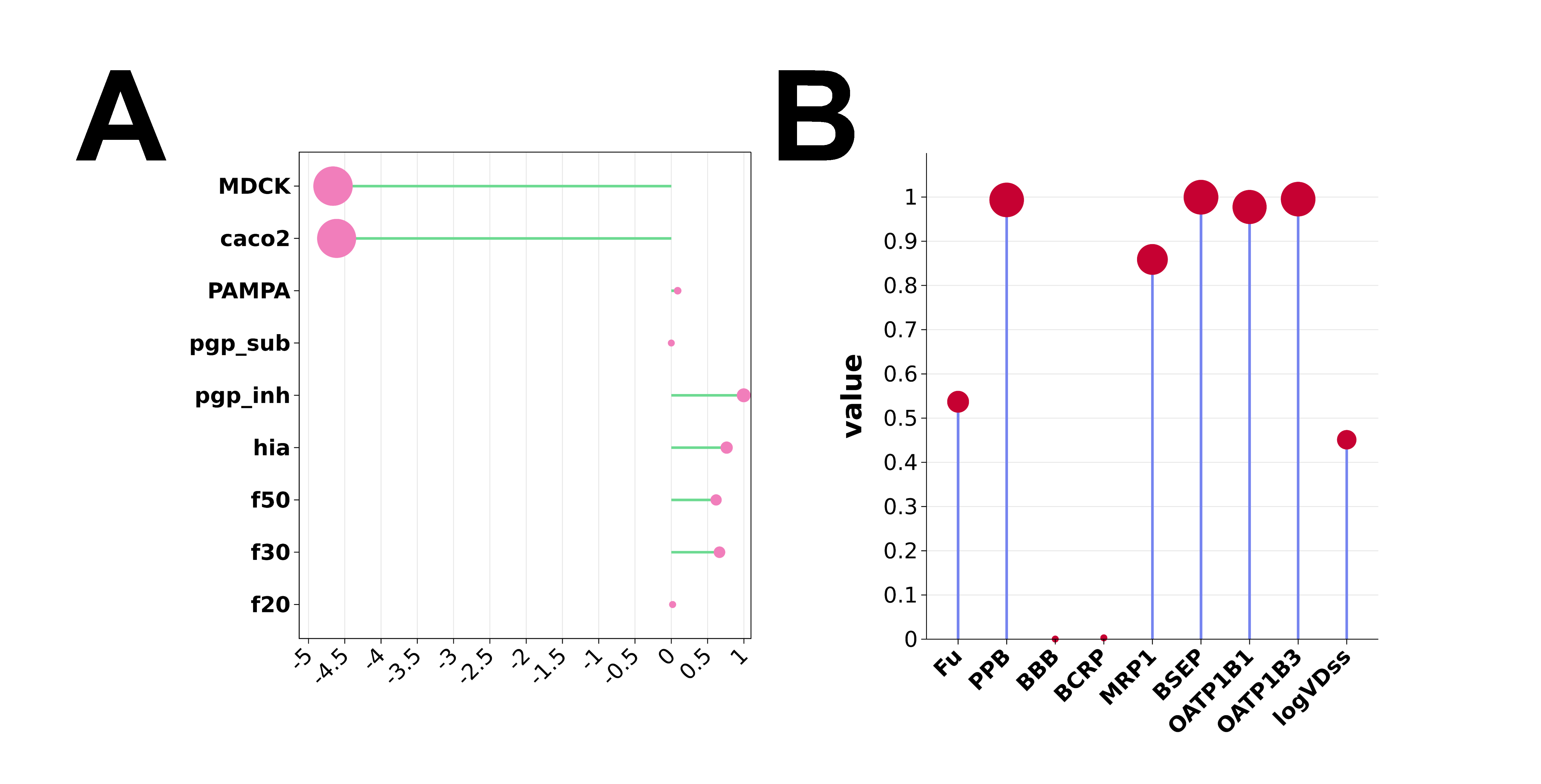

Supplement: Supplementary file 3 [file Image4.TIF]

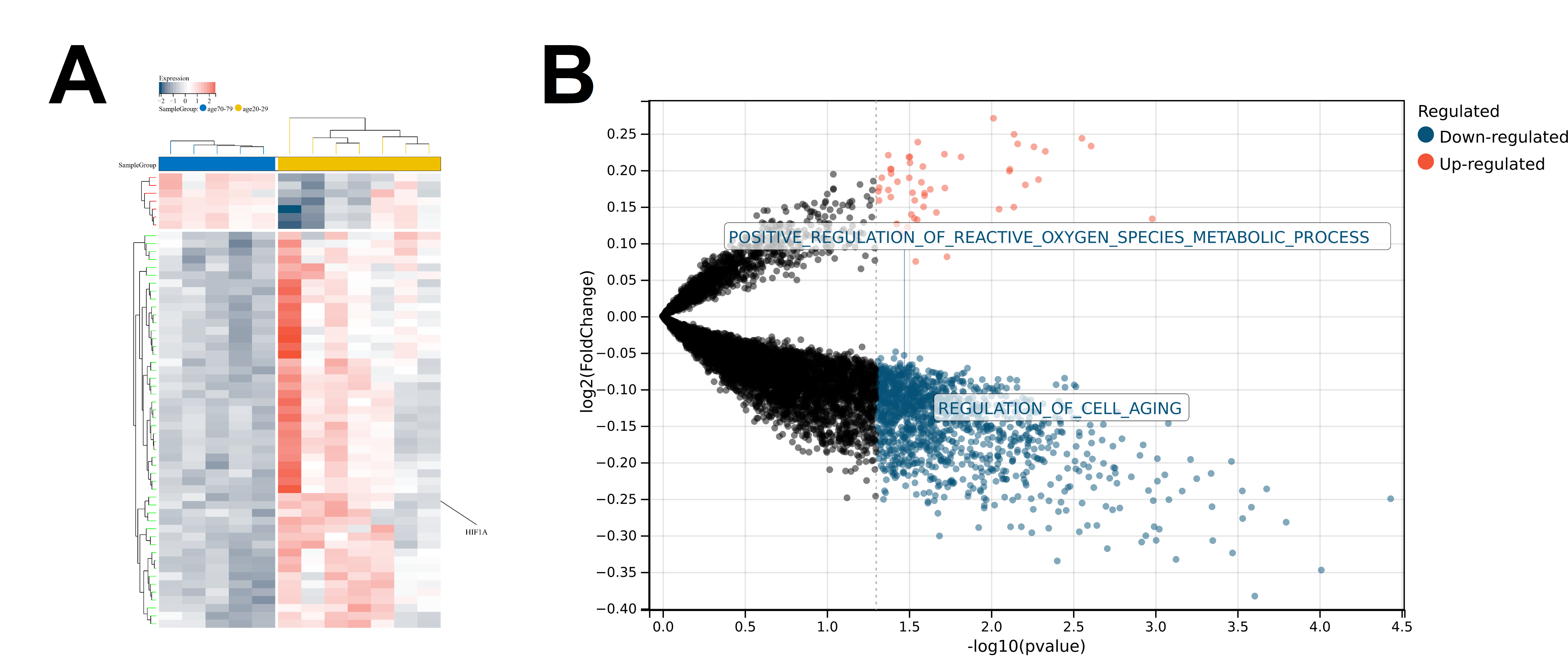

Supplement: Supplementary file 4 [file Image2.TIF]

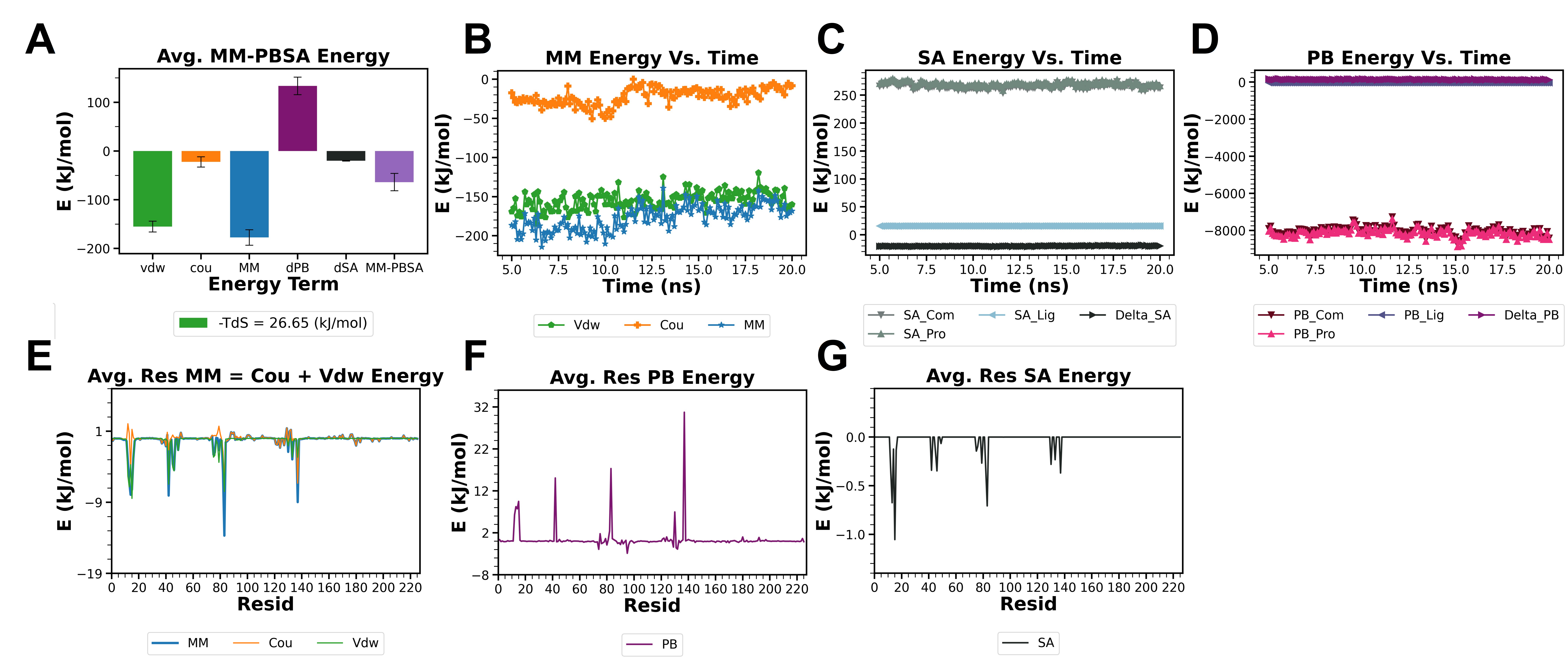

Supplement: Supplementary file 5 [file Image1.TIF]

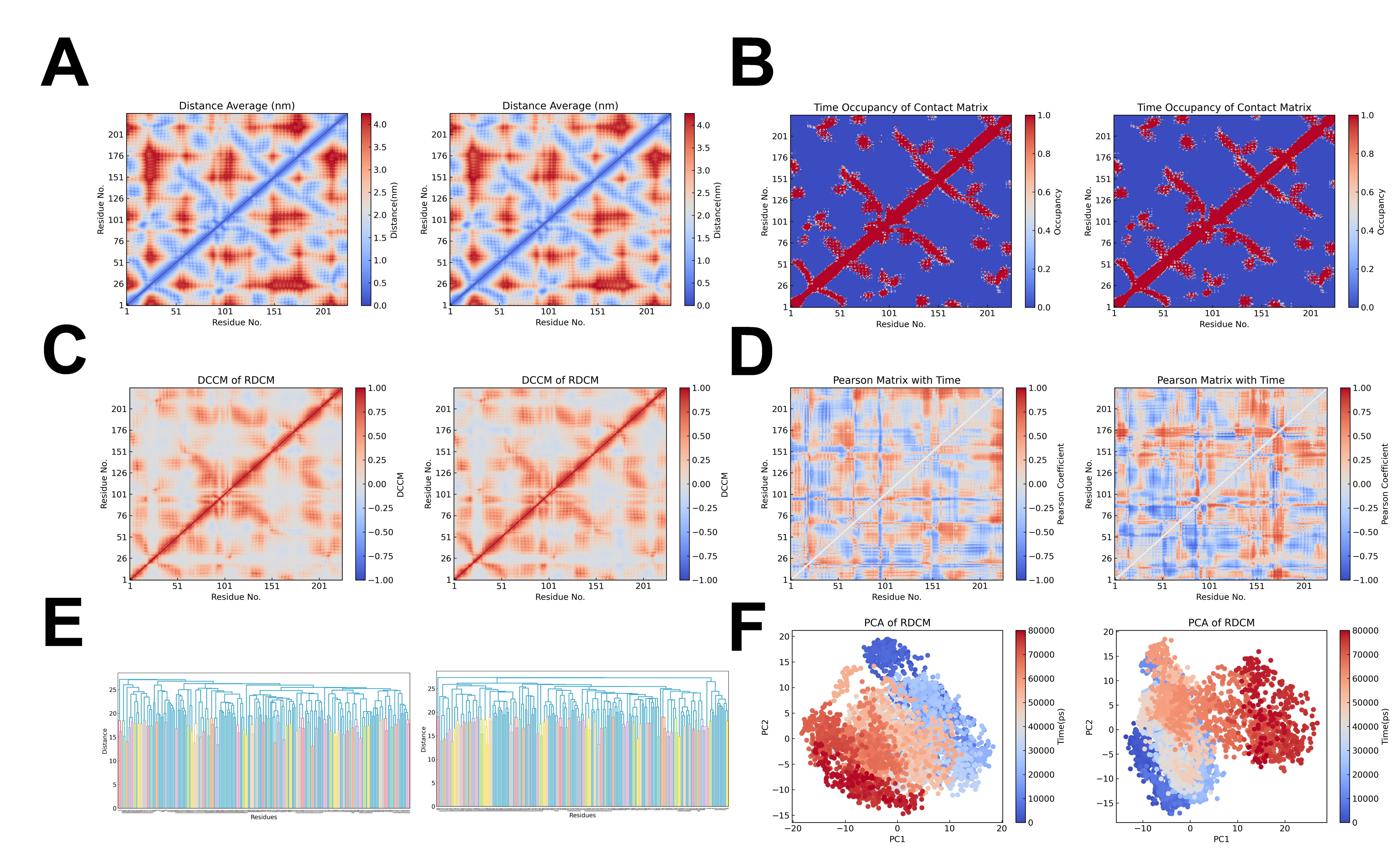

Supplement: Supplementary file 6 [file Image7.TIF]

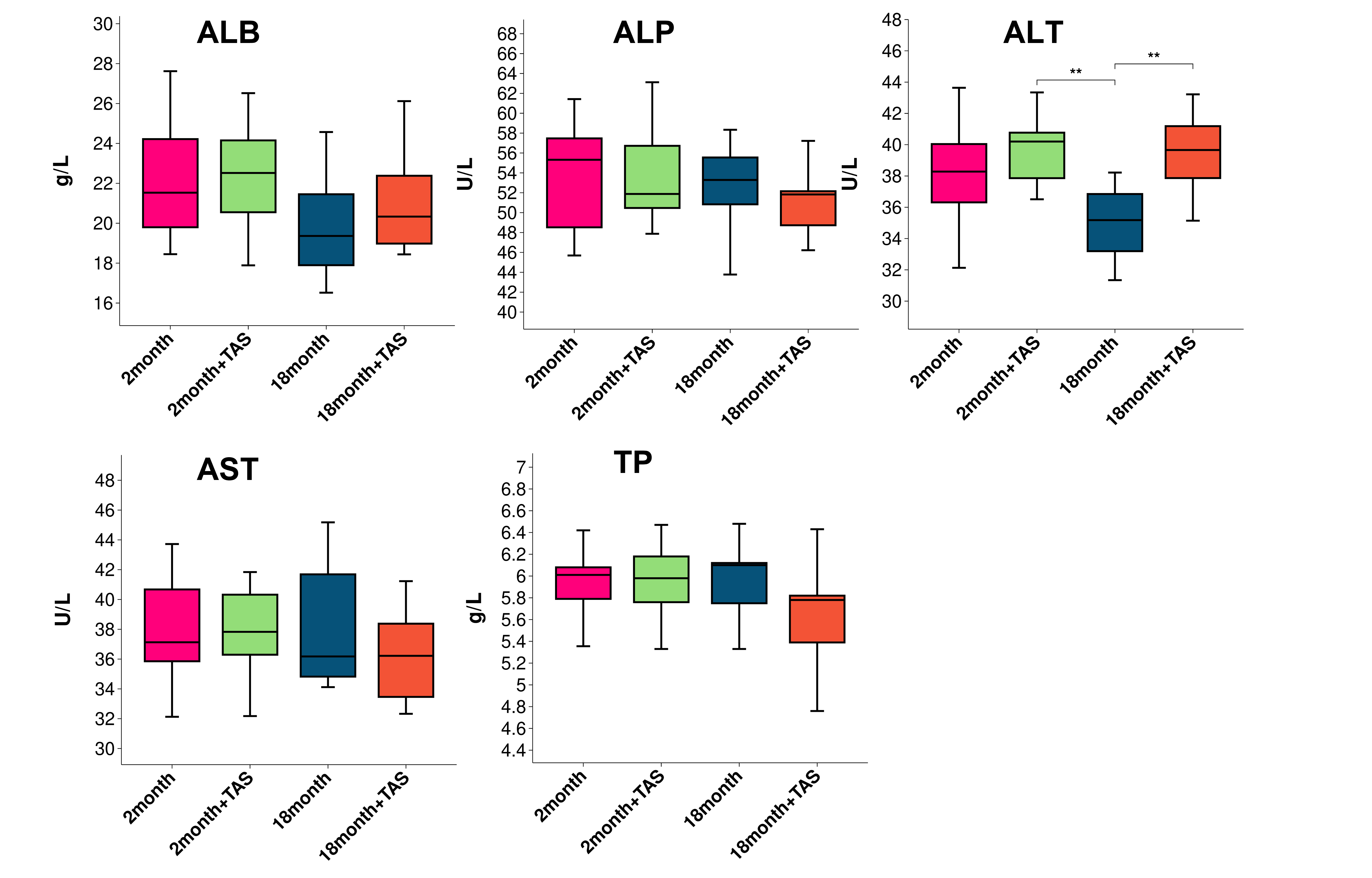

Supplement: Supplementary file 8 [file Image5.TIF]
